# A bimolecular modification strategy for developing long-lasting bone anabolic aptamer

**DOI:** 10.1016/j.omtn.2023.102073

**Published:** 2023-11-10

**Authors:** Huarui Zhang, Sifan Yu, Shuaijian Ni, Amu Gubu, Yuan Ma, Yihao Zhang, Haitian Li, Yuzhe Wang, Luyao Wang, Zongkang Zhang, Yuanyuan Yu, Aiping Lyu, Baoting Zhang, Ge Zhang

**Affiliations:** 1 School of Chinese Medicine, Faculty of Medicine, The Chinese University of Hong Kong, Hong Kong SAR, China; 2 Law Sau Fai Institute for Advancing Translational Medicine in Bone and Joint Diseases, Hong Kong Baptist University, Hong Kong SAR, China; 3 Shenzhen Research Institute, The Chinese University of Hong Kong, Shenzhen, China; 4 Guangdong-Hong Kong-Macao Greater Bay Area International Research Platform for Aptamer-based Translational Medicine and Drug Discovery, Hong Kong SAR, China; 5 The Second Clinical Medical School, Henan University of Chinese Medicine, Zhengzhou, China

**Keywords:** MT: Oligonucleotides: Therapies and Applications, aptamers, long-lasting modification, sclerostin, osteogenesis imperfecta, low-molecular-weight coupling agents

## Abstract

The molecular weight of nucleic acid aptamers (20 kDa) is lower than the cutoff threshold of the renal filtration (30–50 kDa), resulting in a very short half-life, which dramatically limits their druggability. To address this, we utilized 3-(2,5-dioxo-2,5-dihydro-1H-pyrrol-1-yl)-N-(4-hydroxy-2-oxo-2H-chromen-6-yl)propenamide (HC) and 12-((2,5-dioxopyrrolidin-1-yl)oxy)-12-oxododecanoic acid (DA), two newly designed coupling agents, for synergistic binding to human serum albumin (HSA). Both HC and DA are conjugated to a bone anabolic aptamer (Apc001) against sclerostin to form an Apc001OC conjugate with high binding affinity to HSA. Notably, HC and DA could synergistically facilitate prolonging the half-life of the conjugated Apc001 and promoting its bone anabolic potential. Using the designed blocking peptides, the mechanism studies indicate that the synergistic effect of HC-DA on pharmacokinetics and bone anabolic potential of the conjugated Apc001 is achieved via their synergistic binding to HSA. Moreover, biweekly Apc001OC at 50 mg/kg shows comparable bone anabolic potential to the marketed sclerostin antibody given weekly at 25 mg/kg. This proposed bimolecular modification strategy could help address the druggability challenge for aptamers with a short half-life.

## Graphical abstract

**Figure:**
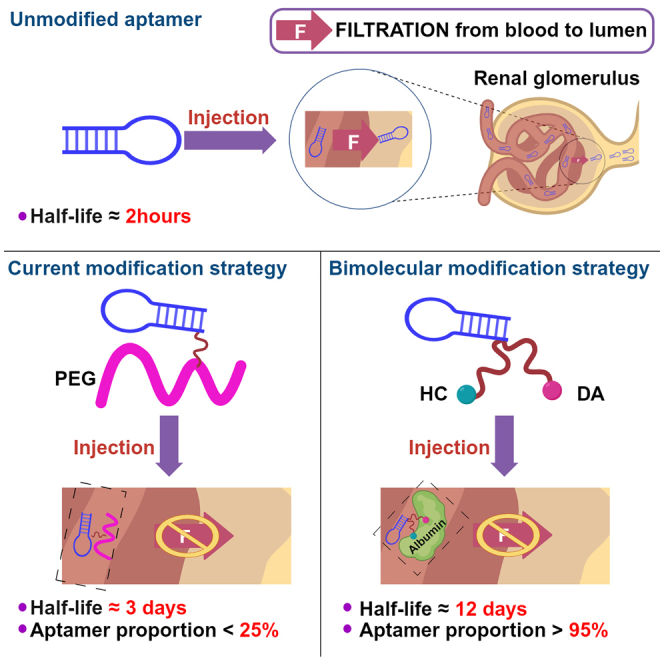

## Teaser

Zhang and colleagues herein developed a bimolecular modification strategy to address the short half-life of unmodified aptamers. This strategy could greatly extend the half-life of the conjugated aptamers (up to 12 days) compared to that of PEGylation (∼3 days), which helps address the druggability challenge for aptamers with a short half-life.

## Introduction

Aptamers are short, single-stranded DNA (ssDNA) or RNA (ssRNA) oligonucleotides, typically composed of 25–80 nucleotides, which can form a range of complex three-dimensional structures. These unique structures enable them to selectively bind to specific targets, including peptides, proteins, small molecules, and even living cells. Remarkably, aptamers can fulfill roles analogous to monoclonal antibodies but offer advantages such as low immunogenicity and ease of high-throughput screening and chemical synthesis when compared to monoclonal antibodies.^1^ The molecular weight of nucleic acid aptamers (20 kDa) is lower than the cutoff threshold of the renal filtration (TVr, 30–50 kDa), resulting in a very short half-life, which dramatically limits their druggability.^1^^,^^2^^,^^3^^,^^4^^,^^5^^,^^6^^,^^7^^,^^8^ The thrombin aptamer NU172 failed in the phase 2 clinical trial due to the short half-life *in vivo* (10 min).^9^ The development of a pharmacokinetics-related aptamer design strategy could help address their druggability challenge. There are some post-selection modification strategies that could prolong the half-life of aptamers.^10^ Accordingly, introducing a bulky moiety is an efficient strategy for averting the rapid renal clearance of aptamers and extending their time in circulation. Among the various substantial functional groups utilized to increase the dimensions of aptamers are polyethylene glycol (PEG), proteins, cholesterol, liposomes, as well as organic and inorganic nanomaterials. Among these choices, PEG has emerged as the predominant option for enhancing the pharmacokinetics attributes and extending the circulation half-life of aptamer candidates *in vivo*.^11^^,^^12^^,^^13^^,^^14^^,^^15^ Riccardi et al. engineered NU172-T^H^9 with an extended *in vivo* half-life of 28.6 h through PEGylation, resulting in enhanced thrombin inhibitory activity.^9^ Nonetheless, the PEG moiety constitutes a substantial portion of the PEG-aptamer conjugate (over 75%). This proportionality poses challenges in augmenting the subcutaneous dosage of the aptamer given a fixed administration volume, significantly restricting its therapeutic potential.^16^^,^^17^^,^^18^^,^^19^ In light of these druggability challenges, it is desirable to seek innovative coupling agents to develop long-lasting therapeutic aptamers with increasable dosages.

Human serum albumin (HSA), the predominant plasma protein (ranging between 35 and 50 g/L in human serum), has a molecular weight of 66.5 kDa.^20^ It is synthesized at an approximate rate of 0.7 mg/h per gram of liver, equating to 10–15 g daily, and exhibits a mean half-life of 19 days.^21^ Strategically, HSA has large hydrophobic interface cages, which could bind to some low-molecular-weight coupling agents (LMWCAs) to form molecular complexes with an average mass above the TVr.^22^ Clinically, several strategies have been adopted to develop long-lasting therapeutics by conjugating a small molecular albumin-binding moiety to either small molecular drugs or peptide drugs.^22^ Mechanistically, LMWCAs can be employed to modify aptamers, producing conjugates that, upon binding to HSA, form molecular complexes with an average mass exceeding the TVr for extending the half-life.^13^ Advantageously, the molecular weight of LMWCAs is substantially lower than that of the aptamer, allowing the aptamer to account for a large proportion of the LMWCA-aptamer conjugate (over 95%). Two reported molecules have strong binding affinity to HSA. One is warfarin and the other is fatty acids (FAs).^23^^,^^24^ However, the anticoagulation effect of warfarin makes it unsuitable to be an LMWCA.^25^^,^^26^ The benzylacetone moiety on warfarin is responsible for this anticoagulant action. Consequently, we designed a novel coumarin derivative (3-(2,5-dioxo-2,5-dihydro-1H-pyrrol-1-yl)-N-(4-hydroxy-2-oxo-2H-chromen-6-yl)propenamide [HC]) exhibiting a potent affinity for HSA without the anticoagulant effect. Besides, octadecanedioic acid (OA) has the strongest binding affinity to HSA among published FAs.^22^^,^^27^^,^^28^^,^^29^^,^^30^^,^^31^ However, OA could also bind to FA-binding proteins (FABPs), which are widely distributed in various tissues, resulting in the decrease of drug amounts in blood circulation.^32^ The length of FA chains could play an important role in the binding to FABP. Thus, we designed a novel FA derivate, 12-((2,5-dioxopyrrolidin-1-yl)oxy)-12-oxododecanoic acid (DA), with low binding affinity to FABP but high binding affinity to HSA.

**Figure 1:**
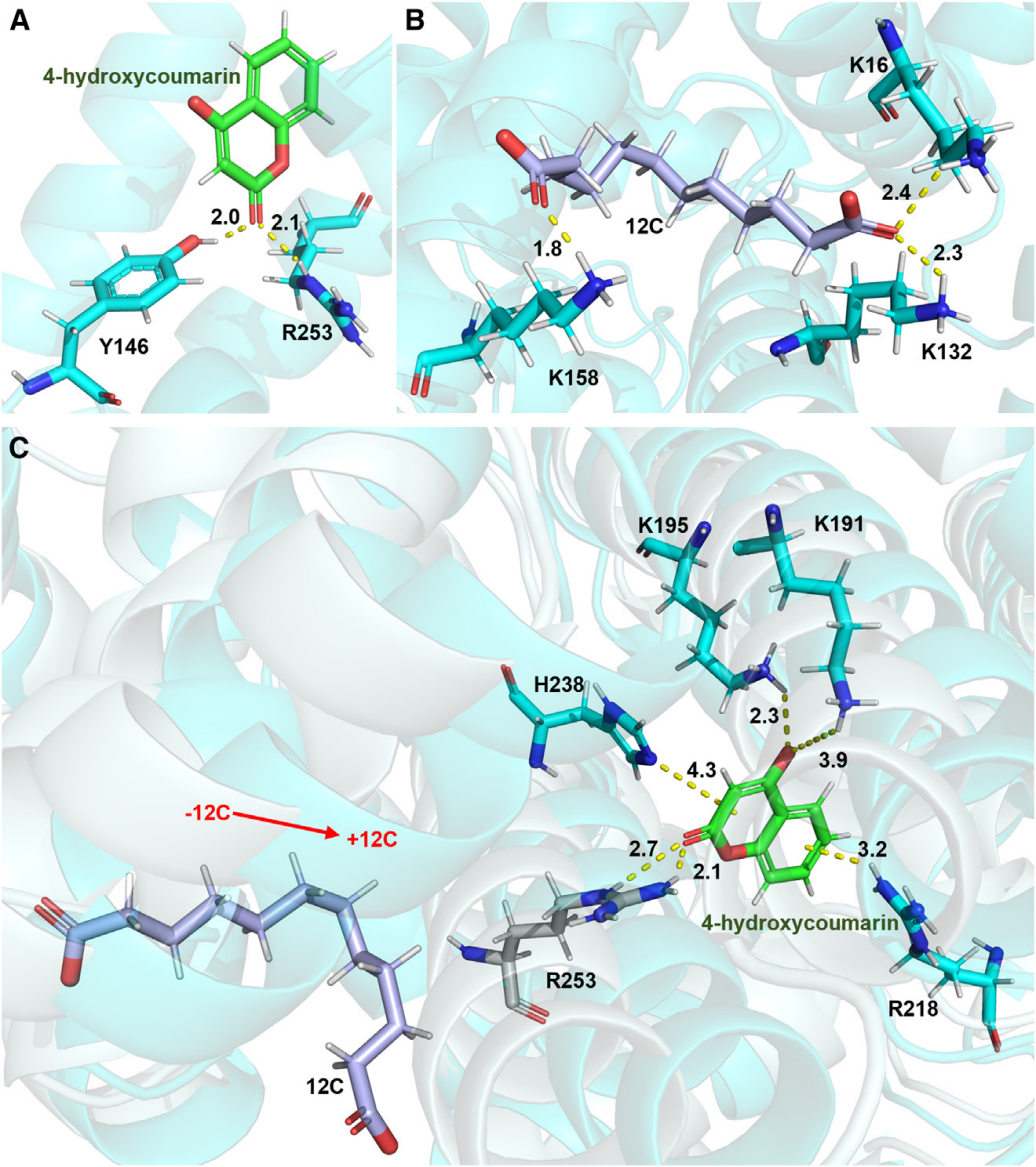
A synergetic interaction between HC and DA to HSA was found by an interaction analysis (A) The predicted interactions between the coumarin moiety of HC (4-hydroxycoumarin, colored in green) and HSA, including two hydrogen bonds with Y146 (2.0 Å) and R253 (2.1 Å), respectively. (B) The predicted interactions between the FA moiety of DA (dodecanedioic acid [12C], colored in light blue) and HSA, including three hydrogen bonds with K16 (2.4 Å), K132 (2.3 Å), and K158 (1.8 Å), respectively. (C) The predicted interactions between the coumarin moiety of HC and HSA, with the presence of the FA moiety of DA near the binding pocket of HC to HSA, including two hydrogen bonds with K191 (3.9 Å) and K195 (2.3 Å), respectively; two cation-π interactions with R218 (3.2 Å) and H238 (4.3 Å), respectively; and two hydrogen bonds with R253 (2.1 and 2.7 Å) alone. The above data predicted that the conformation of the binding pocket of HC to HSA might be significantly altered upon binding of DA, implying a dramatically enhanced binding affinity of HC to HSA. Note: R253 (colored in gray) was the only unaltered predicted binding site between HC and HSA, with or without the presence of 12C. −12C, before molecular dynamic simulation; +12C, after molecular dynamic simulation.

Our previously screened DNA aptamer (Apc001) against sclerostin was proved to exert pro-anabolic potential *in vitro* and bone formation *in vivo*.^12^^,^^33^^,^^34^^,^^35^ Remarkably, the long-lasting sclerostin aptamer for osteogenesis imperfecta (OI) was granted Rare Pediatric Disease Designation (RPD-2022-667) and Orphan Drug Designation (DRU-2022-9087) by the US Food and Drug Administration (FDA) in 2022. In this study, both HC and DA are conjugated to Apc001 to form an Apc001OC conjugate with high binding affinity to HSA. Notably, we found that HC and DA could synergistically facilitate prolonging the half-life of the conjugated Apc001 and promoting its bone anabolic potential. Furthermore, our mechanism studies indicated that the synergistic effect of HC-DA on pharmacokinetics and bone anabolic potential of the conjugated Apc001 is achieved via their synergistic binding to HSA (Figure 1). The proposed innovative long-lasting modification strategy could help in addressing the druggability challenge of aptamers with short half-life.

## Results

### Two novel compounds were designed as candidate LMWCAs with high binding affinity to HSA

The crystal structure of warfarin complexed with HSA has revealed that the interaction between warfarin and HSA appears to be dominated by both hydrophobic contacts and several specific electrostatic interactions.^24^ However, the intrinsic bioactive properties of warfarin pose hemorrhagic concerns in susceptible individuals, rendering warfarin unsuitable for application as a long-acting coupling agent.^25^ The benzylacetone group on warfarin was implicated in decreasing blood clotting ability through the inhibition of vitamin K epoxide reductase, an essential enzyme in the synthesis of vitamin K-dependent coagulation factors.^26^ Consequently, we designed a novel coumarin derivative without anticoagulation effect. A computer-assisted high-throughput virtual screening was employed to identify 4-hydroxycoumarin (integral for HSA binding), exhibiting the highest predicted binding affinity to HSA among over 1,000 coumarin derivatives lacking the benzylacetone group (Figures S1 and S2A).^36^ Given that 4-hydroxycoumarin is unsuitable for direct aptamer conjugation, an aptamer-specific linker was introduced to the coumarin structure to form the candidate LMWCA, HC.

OA holds the strongest binding affinity to HSA among the published FAs.^22^^,^^27^^,^^28^ Besides, it has been reported that the conformation of warfarin-binding pocket in HSA is significantly altered upon FA binding, which dramatically enhances the binding affinity of warfarin to HSA.^29^ However, OA could also bind to FABPs, which are widely distributed in various tissues, resulting in the decrease of drug amounts in blood circulation.^32^ The length of the fatty chains plays an important role in the binding of FAs to FABP.^32^ Therefore, we designed a novel FA derivative with low binding affinity to FABP but high binding affinity to HSA. Virtual molecular docking identified the dodecanedioic acid (the key binding structure to HSA) with the lowest predicted binding affinity to FABP but comparably high predicted binding affinity to HSA from a series of FA derivatives varying in length and side-chain modifications (Figure S2B). To transform dodecanedioic acid into a suitable coupling agent for aptamer conjugation, an aptamer-specific linker was introduced to the FA structure, generating the agent DA.

### The synthesis of DA and HC

**Figure 2:**
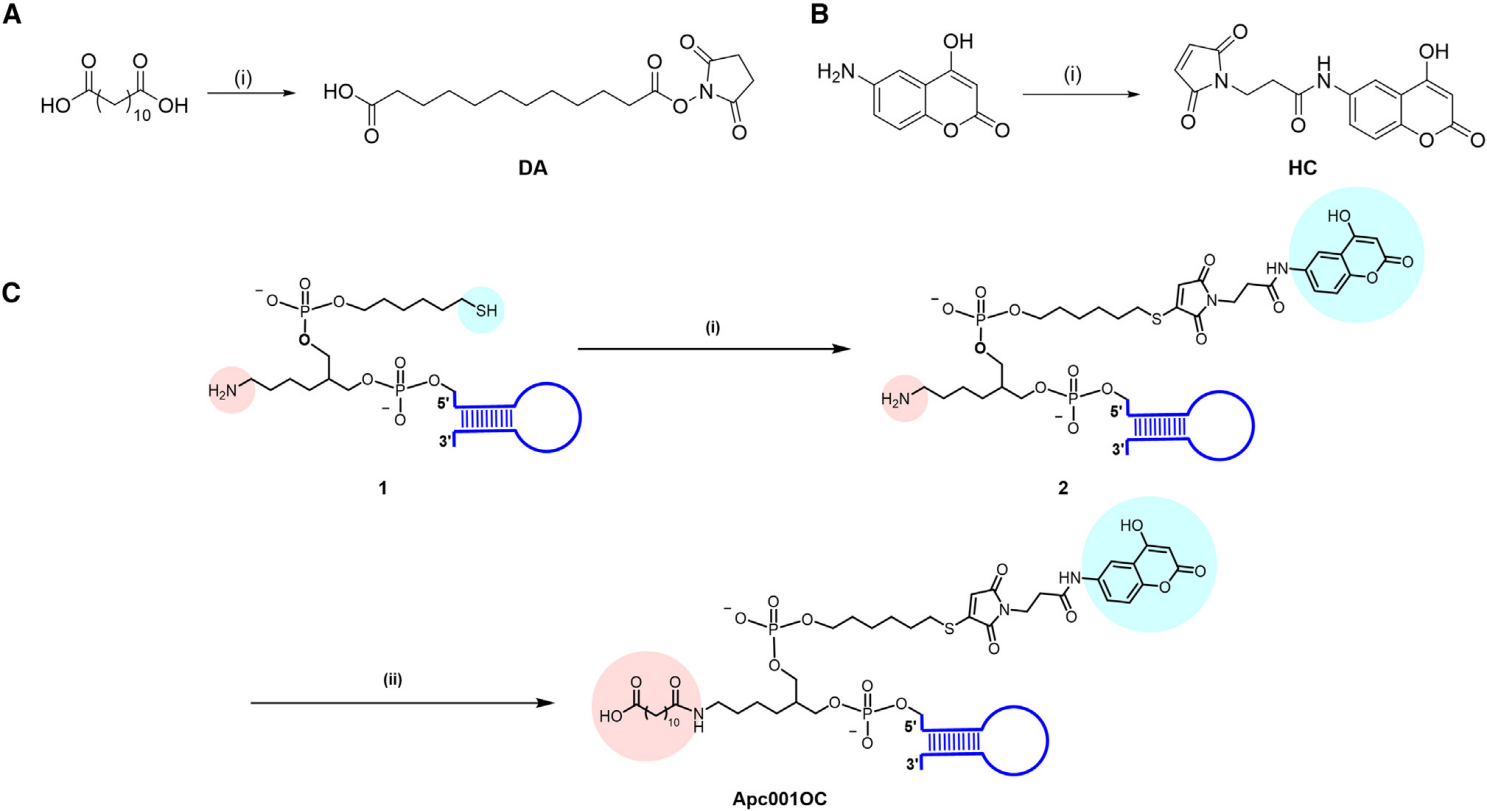
Synthesis of key intermediates and the conjugation of HC and DA to Apc001 (A) The synthesis route of DA. Note for reagents and conditions: (i) 1-hydroxypyrrolidine-2, 5-dione, DCC, 4-DMAP, room temperature (RT), THF. (B) The synthesis route of HC. Note for reagents and conditions: (i) 3-(2,5-dioxo-2,5-dihydro-1H-pyrrol-1-yl)propanoic acid, HBTU, DIPEA, RT, DMF. (C) Synthesis of Apc001OC. Note for reagents and conditions: (i) HC, H_2_O/DMF, pH = 6.75; (ii) DA, H_2_O/DMF, pH = 8.4.

Synthesis of DA (Figure 2A) commenced with dodecanedioic acid and 1-hydroxypyrrolidine-2,5-dione in the presence of dicyclohexylcarbodiimide (DCC) and N,N-dimethylaminopyridine (DMAP). To synthesize HC (Figure 2B), 6-amino-4-hydroxy-2H-chromen-2-one was adding into the N,N-dimethylformamide (DMF) solution of 3-(2,5-dioxo-2,5-dihydro-1H-pyrrol-1-yl)propanoic acid with the presence of hexafluorophosphate benzotriazole tetramethyl uronium (HBTU) and N,N-diisopropylethylamine (DIPEA). The structures of both DA and HC were confirmed by ^1^H NMR, ^13^C NMR, and high-resolution mass spectrometry (Figures S11 and S12).

### Experimental validation of HC and DA as candidate LMWCAs with high binding affinity to HSA

To validate the binding affinity of HC to HSA, surface plasmon resonance (SPR) assay was performed. Our data demonstrated that HC had high binding affinity to HSA (dissociation constant [K_d_] = 6.54 nM) (Figure S2A). To validate the abolished anticoagulant effect of HC, the anticoagulant assay was performed. The anticoagulant assay showed that warfarin markedly reduced the clotting time, whereas HC had no anticoagulant effect (Figure S2A). As shown by the above data from both SPR assay and anticoagulant assay, HC could demonstrate high binding affinity to HSA but without anticoagulant effect.

To validate the binding affinity of DA to HSA and FABP, respectively, SPR assay was performed. Our data demonstrated that DA had low binding affinity to FABP (K_d_ = 90.64 nM) but still retained high binding affinity to HSA (K_d_ = 40.52 nM) (Figures S2C and S2D). Moreover, the binding affinity of DA to FABP was significantly lower than that of OA to FABP (K_d_ = 8.62 nM). As shown by the above data from the SPR assay, DA could demonstrate low binding affinity to FABP but high binding affinity to HSA.

### Synthesis of Apc001OC conjugate

**1** (amino-mercapto-modified Apc001 [Apc001-SH-NH_2_]) was conjugated with both HC and DA to form Apc001OC. The detailed synthesis route of Apc001OC is described below. Synthesis of Apc001OC (Figure 2C) commenced with **1** and HC in the mixture of double-distilled H_2_O (ddH_2_O) and DMF (pH = 6.75) to provide **2**. Without further purification, **2** was reacted with DA in the presence of NaHCO_3_, providing Apc001OC. Apc001-SH was conjugated with HC to form Apc001HC (Figure S3A). Amino-modified Apc001 (Apc001-NH_2_) was conjugated with DA to form Apc001DA (Figure S3B). The structures of key intermediates were confirmed by high-performance liquid chromatography (HPLC) and mass spectrometry (Figures S13–S15). Apc001OC was characterized by HPLC and mass spectrometry (Figure S16). The structures of Apc001HC and Apc001DA were confirmed by HPLC and mass spectrometry (Figures S17 and S18).

### Both HC and DA could synergistically facilitate the binding affinity of the conjugated Apc001 to HSA

**Figure 3:**
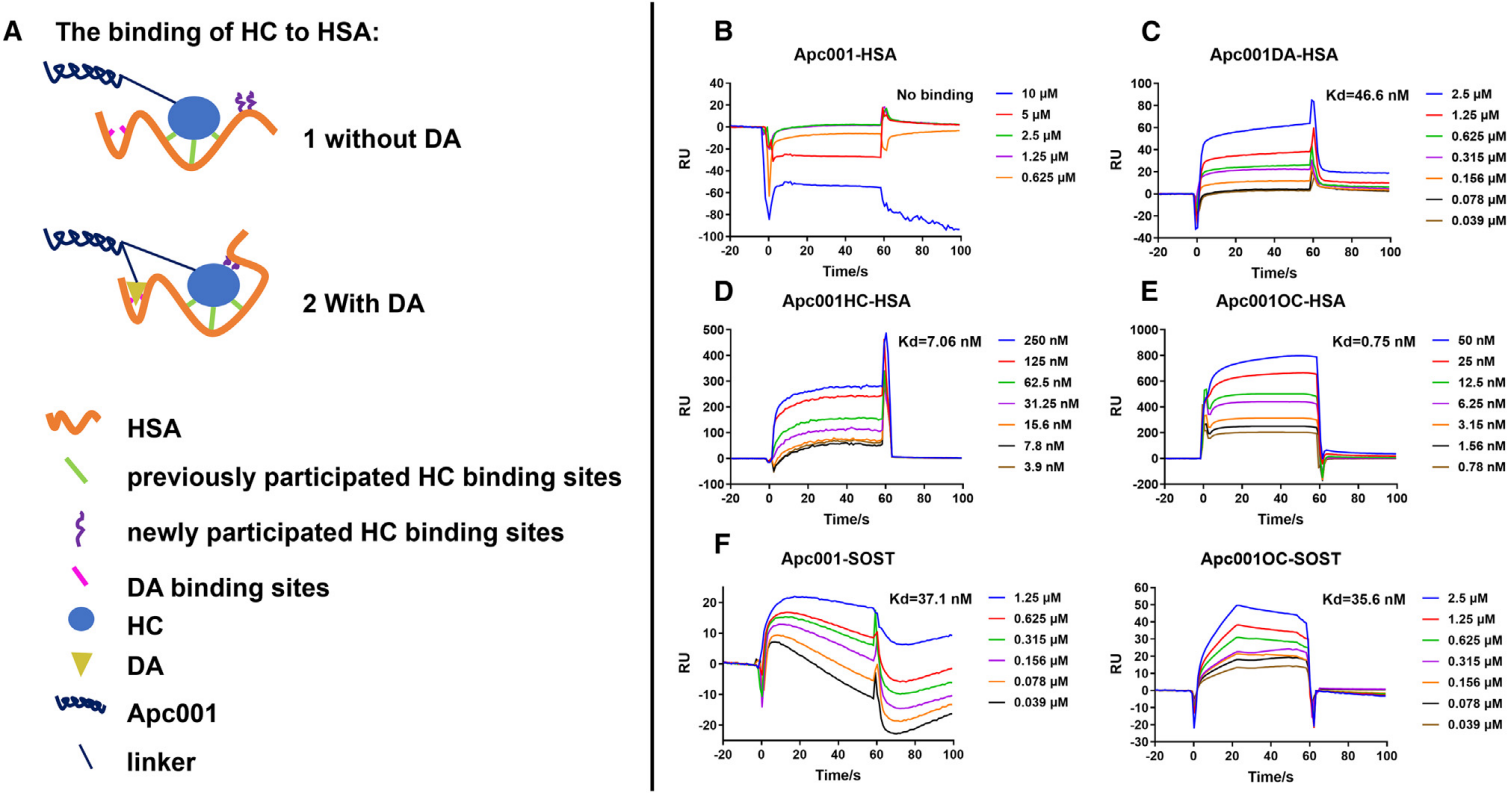
Both HC and DA were conjugated to a bone anabolic aptamer against sclerostin (Apc001) to form an Apc001OC conjugate with a high binding affinity to HSA (A) Schematic diagram of the binding mode of Apc001OC to HSA. (B) The multi-cycle kinetics analysis of binding affinity of Apc001 to HSA. (C) The multi-cycle kinetics analysis of binding affinity of Apc001DA to HSA (K_d_ = 46.6 nM). (D) The multi-cycle kinetics analysis of binding affinity of Apc001HC to HSA (K_d_ = 7.06 nM). (E) The multi-cycle kinetics analysis of binding affinity of Apc001OC to HSA (K_d_ = 0.75 nM). (F) Left: the multi-cycle kinetics analysis of binding affinity of Apc001 to sclerostin (SOST) (K_d_ = 37.1 nM). Right: the multi-cycle kinetics analysis of binding affinity of Apc001OC to SOST (K_d_= 35.6 nM).

The binding affinity of Apc001OC, Apc001HC, and Apc001DA to HSA was analyzed by SPR. Our data demonstrated that the Apc001OC conjugate (K_d_ = 0.75 nM) had dramatically stronger binding affinity to HSA than Apc001HC (K_d_ = 7.06 nM) and Apc001DA (K_d_ = 46.6 nM). A synergetic interaction mechanism between HC and DA was predicted using a binding-mode-based interaction analysis by molecular dynamic simulation (Figures 1 and 3A–3E). Without the presence of DA near the binding pocket of HC to HSA, there were two predicted interactions between HC and HSA, including two hydrogen bonds with Y146 (2.0 Å) and R253 (2.1 Å), respectively (Figure 1A). When DA bound HSA alone, there were three predicted interactions between DA and HSA, including three hydrogen bonds with K16 (2.4 Å), K132 (2.3 Å), and K158 (1.8 Å), respectively (Figure 1B). Notably, with the presence of DA near the binding pocket of HC to HSA, there were six predicted interactions between HC and HSA, including two hydrogen bonds with K191 (3.9 Å) and K195 (2.3 Å), respectively; two cation-π interactions with R218 (3.2 Å) and H238 (4.3 Å), respectively; and two hydrogen bonds with R253 (2.1 Å, 2.7 Å) alone (Figure 1C). To efficiently demonstrate the increased binding affinities of different Apc001 conjugates to HSA, we normalized the binding affinity of Apc001DA to HSA (K_d_ = 46.6 nM) as 100%. As a result, the binding affinity of Apc001HC to HSA (K_d_ = 7.06 nM) showed an increase of 660% compared to the binding affinity of Apc001DA to HSA. More importantly, the binding affinity of Apc001OC to HSA (K_d_ = 0.75 nM) had an increase of 6,200% compared to the binding affinity of Apc001DA to HSA (Table S1). This means that the increase in the binding affinity of Apc001OC to HSA was dramatically higher than the sum of the increases in the binding affinity of Apc001HC to HSA and Apc001DA to HSA (6,200% > 760%, where 760% = 660% + 100%). Therefore, we concluded that HC and DA could synergistically facilitate the binding affinity of the conjugated Apc001 to HSA.

To elucidate the binding affinity of the conjugated Apc001 to sclerostin, SPR assay was performed. Our data demonstrated that the Apc001OC conjugate (K_d_ = 35.6 nM) had a comparable binding affinity to sclerostin when compared to Apc001 (K_d_ = 37.1 nM) (Figure 3F), suggesting that the introduction of both HC and DA did not perturb the inherent affinity of Apc001 to sclerostin.

### Four blocking peptides were designed to block the binding of HC, DA, and HC-DA combination and HC-DA interaction to HSA, respectively

The coumarin structure could contribute to the high binding affinity to HSA.^24^ Based on the calculated binding sites of HSA to the coumarin moiety of HC, it was predicted that Y146 and R253 on HSA were involved. Consequently, a peptide (peptide A) including these residues was designed and its binding affinity to HC was validated via SPR (K_d_ = 15.56 nM) (Figure S4A). The FA structure could contribute to the high binding affinity to HSA.^22^^,^^27^^,^^28^ Based on the calculated binding sites of HSA to the FA moiety of DA, it was predicted that K16, K132, and K158 on HSA were involved.^37^ A corresponding peptide (peptide B) including the above binding sites was designed and its affinity to DA was validated by SPR (K_d_ = 40.15 nM) (Figure S4B). Based on the above prediction, a peptide sequence including the above binding sites that could bind both HC and DA was designed (peptide C). Then we verified its binding affinity to both HC and DA by SPR analysis (K_d_ = 13.78 nM for HC, K_d_ = 30.62 nM for DA) (Figure S4C). Furthermore, based on the calculated binding sites of HSA to HC in the presence and absence of DA (Figure S5A), it was predicted that residues Y146 and R253 on HSA were involved in the absence of DA, while, in its presence, residues K191, K195, R253, R238, and H238 on HSA were involved in the presence of DA. Of note, K191, K195, R238, and H238 were the newly participated binding sites in the presence of DA (Figure S5B). Then, a peptide sequence (peptide D) including the above newly participated binding sites was designed and its affinity to HC was validated by SPR (K_d_ = 26.82 nM) (Figure S5C). We designed the negative peptide sequences (NC1 for peptide A, NC2 for peptide B, NC3 for peptide C, NC4 for peptide D) by mutating the predicted binding amino acids sites to alanine on each blocking peptide.

### The binding affinity of Apc001OC conjugate to HSA was significantly reduced or abolished when it was pre-saturated with our designed blocking peptide sequences

The above data provided the following validated research tools: peptide A (designed to block the binding of HC to HSA), peptide B (designed to block the binding of DA to HSA), peptide C (designed to block the binding of HC-DA combination to HSA), and peptide D (designed to block the binding of HC-DA interaction to HSA). Mechanistically, the binding affinity of the Apc001OC conjugate to HSA was significantly reduced when it was pre-saturated with either peptide A (from K_d_ = 0.75 nM to K_d_ = 27.8 nM) or peptide B (from K_d_ = 0.75 nM to K_d_ = 23.5 nM) (Figures S6A and S6B). The binding affinity was abolished when it was pre-saturated with peptide C (Figure S6C). Together, our data indicated the important roles of HC, DA, and HC-DA combination in Apc001OC conjugate binding to HSA, respectively. Moreover, both HC and DA could synergistically facilitate the binding affinity of the conjugated Apc001 to HSA. Importantly, the synergistically enhanced binding affinity of the conjugated Apc001 to HSA by HC and DA was abolished when pre-saturated with peptide D (from K_d_ = 0.75 nM to K_d_ = 12.61 nM) (Figure S6D). Our findings indicated that DA could alter the conformation of the HC binding pocket in HSA and drive more amino acid residues to bind to HC, including K191, K195, R238, and H238, which could explain the contribution of the interaction between HC and DA to the synergistic effect.

### HC and DA could synergistically prolong the half-life of the conjugated Apc001 toward 289.94 h in normal rats

**Figure 4:**
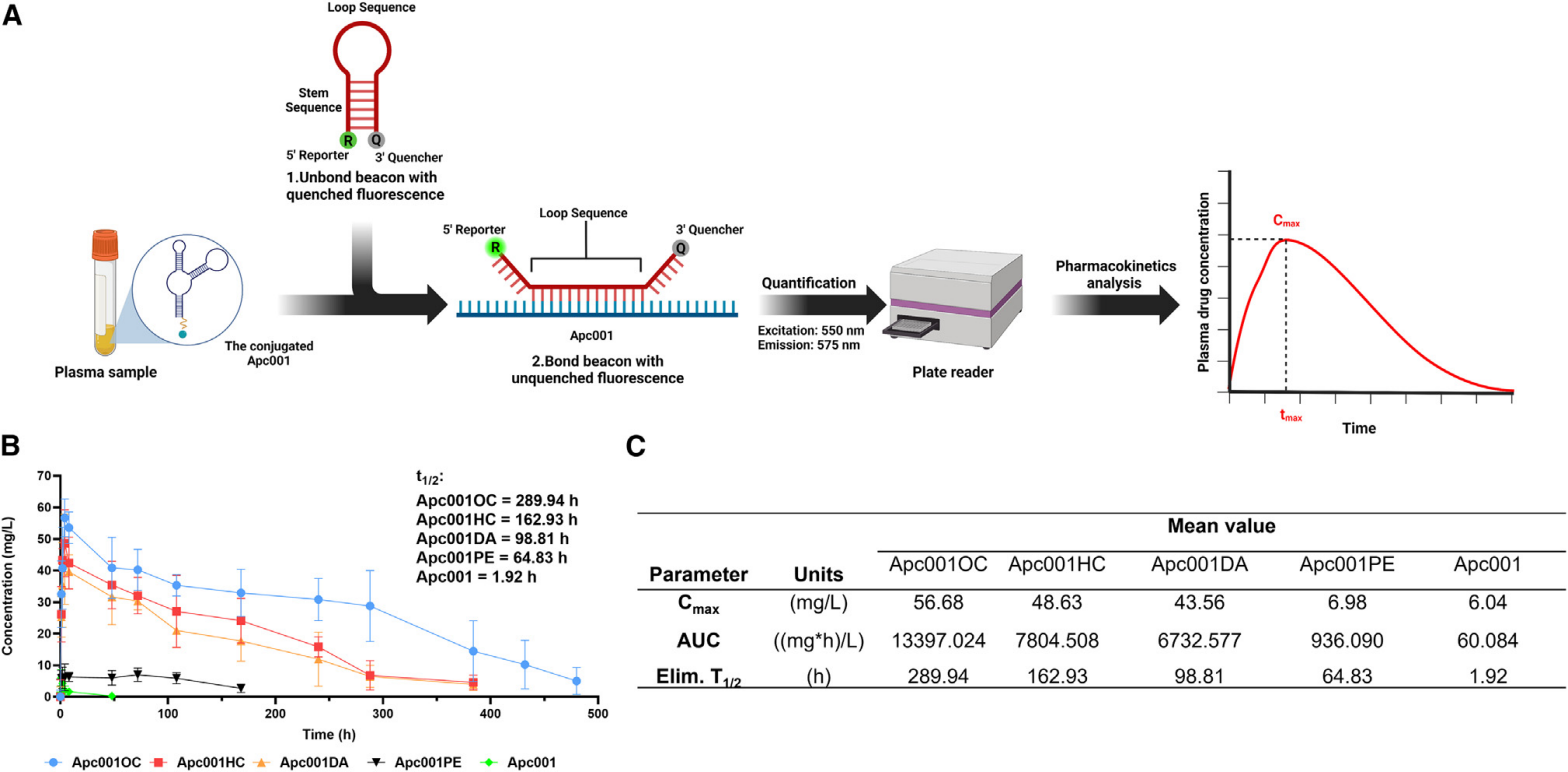
HC and DA could synergistically facilitate prolonging the circulation half-life of the conjugated Apc001 in normal rats (A) A diagram showing the quantification of the amount of the conjugated Apc001 from the blood. (B) Pharmacokinetics of a single subcutaneous (s.c.) injection of Apc001OC (6.25 mg/kg), Apc001HC (6.25 mg/kg), Apc001DA (6.25 mg/kg), Apc001PE conjugate (6.25 mg/kg), and Apc001 (6.25 mg/kg) in normal rats. The Apc001OC conjugate (t_1/2_ = 289.94 h) had a longer half-life compared with the Apc001HC conjugate (t_1/2_ = 162.93 h) and Apc001DA conjugate (t_1/2_ = 98.81 h). (C) Pharmacokinetic parameters of Apc001OC, Apc001HC, Apc001DA, Apc001PE, and Apc001 administered s.c., respectively. t_1/2,_ half-life.

From our pharmacokinetics studies in normal rats, a single subcutaneous administration of Apc001OC conjugate (6.25 mg/kg), Apc001HC conjugate (6.25 mg/kg), Apc001DA conjugate (6.25 mg/kg), PEG40-modified Apc001 (Apc001PE) (6.25 mg/kg), and Apc001 (6.25 mg/kg) was performed.^12^^,^^33^ The plasma concentrations of the aptamers at each time point after the subcutaneous administration were analyzed by molecular beacon method (Figure 4A). The Apc001OC conjugate (t_1/2_ = 289.94 h) had a longer half-life compared with Apc001HC conjugate (t_1/2_ = 162.93 h), Apc001DA conjugate (t_1/2_ = 98.81 h), and Apc001PE conjugate (t_1/2_ = 64.83 h) (Figure 4B). The half-life of the non-conjugated Apc001 was only 1.92 h (Figure 4B). Moreover, the Apc001OC conjugate (AUC_0-t_ = 13,397 (mg∗h)/L) had a lower clearance rate in comparison to Apc001HC conjugate (AUC_0-t_ = 7,804.508 (mg∗h)/L), Apc001DA conjugate (AUC_0-t_ = 6,732.577 (mg∗h)/L), Apc001PE conjugate (AUC_0-t_ = 936.090 (mg∗h)/L), and Apc001 conjugate (AUC_0-t_ = 60.084 (mg∗h)/L) (Figure 4C). The t_1/2_ value of 289.94 h could afford an interval of 2 weeks at most for the administration of the Apc001OC conjugate, whereas the t_1/2_ value of 56 h could only afford an interval of 3 days at most for romosozumab.^38^^,^^39^ Similarly, the t_1/2_ value of 64.83 h could only afford an interval of 3 days at most for Apc001PE. It suggested that the half-life of the Apc001OC conjugate in normal rats was at least four times longer than that of both romosozumab and Apc001PE. More importantly, the half-life of the Apc001OC conjugate (t_1/2_ = 289.94 h) was even longer than the addition of the half-life of the Apc001HC conjugate (t_1/2_ = 162.93 h) and the half-life of the Apc001DA conjugate (t_1/2_ = 98.81 h). Our findings indicated that both HC and DA could synergistically facilitate prolonging the circulation half-life of the conjugated Apc001 in normal rats.

### The HC-DA combination effect on prolonging the half-life of the conjugated Apc001 was reduced or abolished when it was pre-saturated with our designed blocking peptide sequences in normal rats

From the above binding validation data, peptide A, peptide B, and peptide C could block the binding of HC, DA, and HC-DA combination to HSA, respectively. Then, the normal rats were subcutaneously administered with Apc001OC, which is pre-saturated by different blocking peptides (denoted as peptide A/B/C@Apc001OC) or these blocking peptides alone, respectively, to investigate the role of HC-DA combination in prolonging the half-life. The half-life of the different blocking peptides was less than 1 h (Figure S21). Our data demonstrated that the HC-DA combination effect on prolonging the half-life was reduced when it was pre-saturated with either peptide A (from t_1/2_ = 289.94 h to t_1/2_ = 96.3 h) or peptide B (from t_1/2_ = 289.94 h to t_1/2_ = 170.1 h) (Figure S7). Importantly, the HC-DA combination effect on prolonging the half-life was abolished when it was pre-saturated with peptide C (from t_1/2_ = 289.94 h to t_1/2_ = 6.4 h)^12^^,^^33^ (Figure S7), indicating the indispensable contributions of both HC and DA in Apc001OC conjugate to synergistically facilitate prolonging the circulation half-life of the conjugated Apc001.

### The HC-DA interaction effect on prolonging the half-life of the conjugated Apc001 was abolished when it was pre-saturated with peptide D in normal rats

From the above binding validation data, the peptide D could block the binding of HC-DA interaction to HSA. Then, the normal rats were subcutaneously administered with Apc001OC, which is pre-saturated by peptide D (denoted as peptide D@Apc001OC) to investigate the role of HC-DA interaction in prolonging the half-life. Our findings demonstrated that the HC-DA interaction effect on prolonging the half-life was abolished when it was pre-saturated with peptide D (from t_1/2_ = 289.94 h to t_1/2_ = 196.8 h) (Figure S7), indicating the important role of the HC-DA interaction in the Apc001OC conjugate in synergistically prolonging the circulation half-life of the conjugated Apc001.

### HC and DA could synergistically facilitate the conjugated Apc001, promoting bone formation, increasing bone mass, and improving bone microarchitecture integrity in *Col1a2*^*+/G610C*^ mice

**Figure 5:**
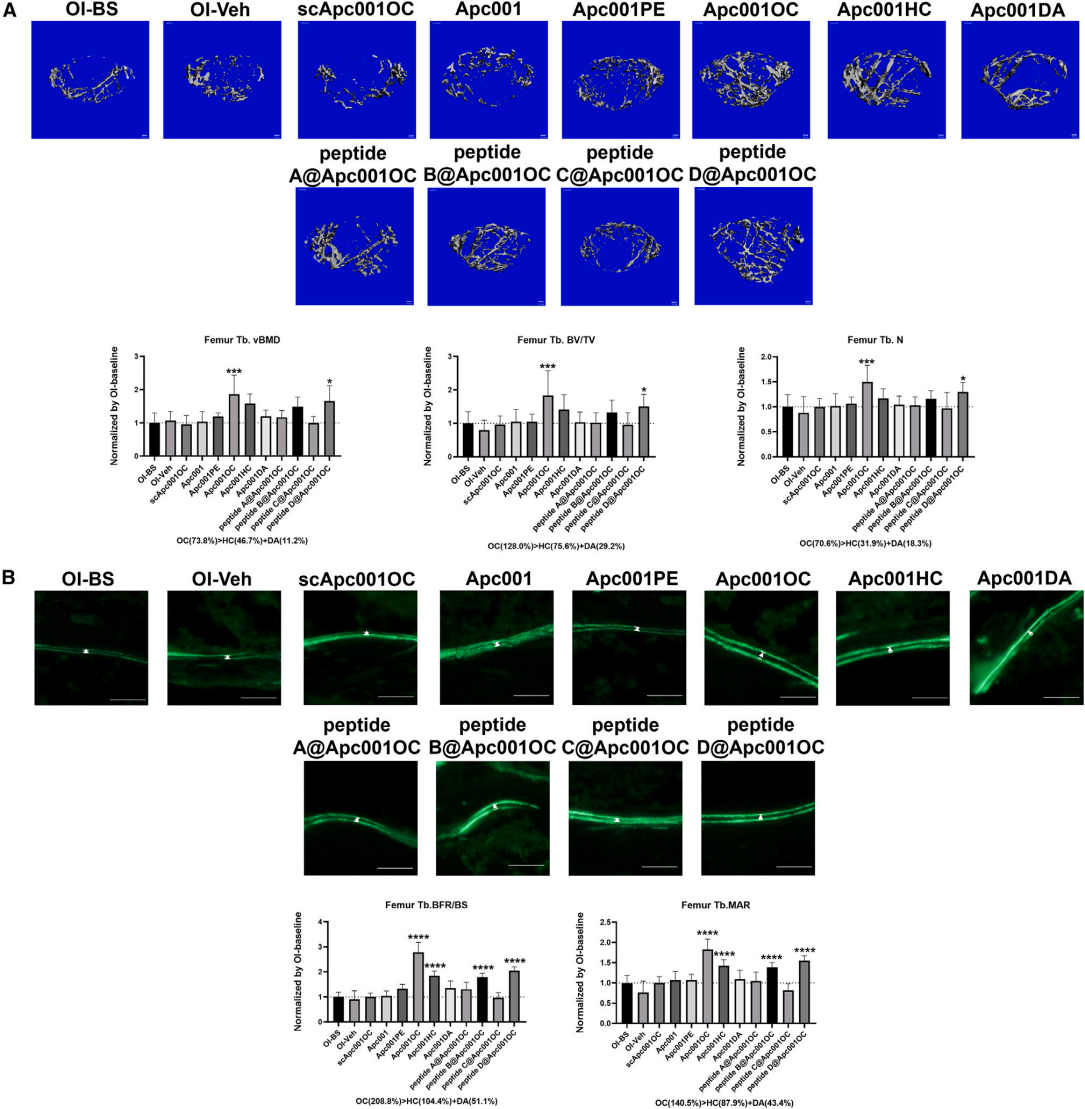
HC and DA could synergistically facilitate the conjugated Apc001 promoting bone formation, increasing bone mass, and improving bone microarchitecture integrity in OI mice (A) Representative images showing three-dimensional trabecular architecture by micro-CT reconstruction at the distal femur. Scale bars, 0.1 mm (the upper panel). Bar charts of the structural parameters of Tb.vBMD, Tb.BV/TV, and Tb.N from *ex vivo* micro-CT examination at the distal femur for the OI mice injected subcutaneously with 25 mg/kg of Apc001OC conjugate, Apc001HC conjugate, Apc001DA conjugate, scApc001OC conjugate, Apc001PE conjugate, Apc001, peptide A@Apc001OC, peptide B@Apc001OC, peptide C@Apc001OC, or peptide D@Apc001OC once every 2 weeks (q2wk) for 12 weeks, respectively (the lower panel). (B) Representative fluorescent micrographs of the trabecular bone sections showing bone formation at the distal femur visualized by double calcein labels. Scale bars, 30 μm (the upper panel). Analysis of dynamic bone histomorphometric parameters of Tb.BFR/BS and Tb.MAR at the distal femur for the OI mice (the lower panel). BS, baseline; Veh, vehicle; Peptide A/B/C/D@Apc001OC, Apc001OC that is pre-saturated by different blocking peptides; peptide A, a peptide sequence within HSA including the predicted binding sites that could bind HC; peptide B, a peptide sequence within HSA including the predicted binding sites that could bind DA; peptide C, a peptide sequence within HSA including the predicted binding sites that could bind both HC and DA; peptide D, a peptide sequence within HSA including the predicted newly participated binding sites that could bind HC with the presence of DA. Data are expressed as mean ± standard deviation followed by one-way ANOVA with Tukey’s *post hoc* test, n = 6 per group. ∗p < 0.05, ∗∗p < 0.01, ∗∗∗p < 0.005, ∗∗∗∗p < 0.0001.

**Figure 6:**
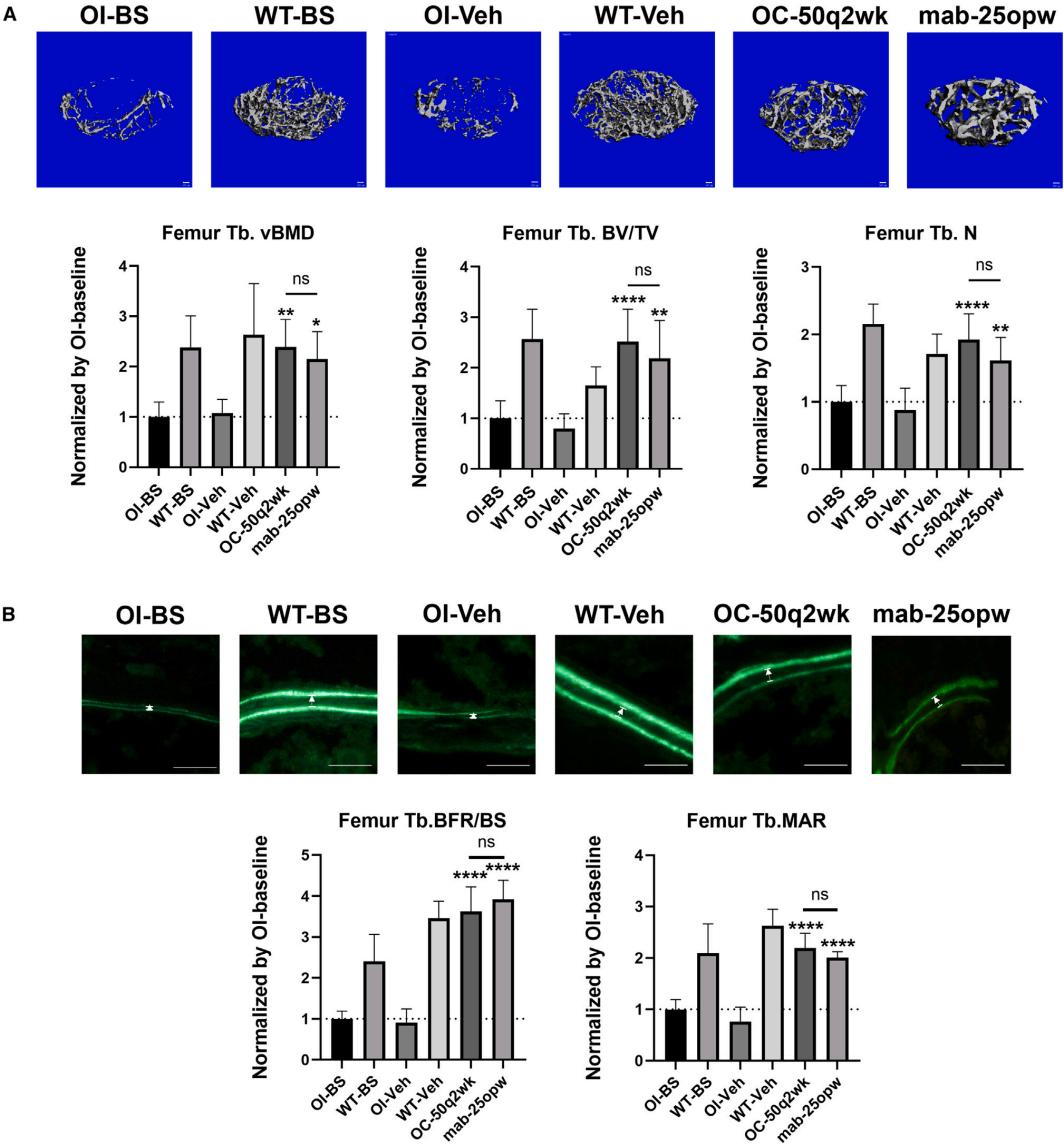
Apc001OC with 50 mg/kg q2wk showed comparable effect on promoting bone anabolic potential at distal femur in OI mice when compared to sclerostin antibody with 25 mg/kg opw (A) Representative images showing three-dimensional trabecular architecture by micro-CT reconstruction at the distal femur. Scale bars, 0.1 mm (the upper panel). Bar charts of the structural parameters of Tb.vBMD, Tb.BV/TV, and Tb.N from *ex vivo* micro-CT examination at the distal femur for the OI mice injected subcutaneously with 50 mg/kg of Apc001OC conjugate q2wk or 25 mg/kg of romosozumab for 12 weeks, respectively (the lower panel). (B) Representative fluorescent micrographs of the trabecular bone sections showing bone formation at the distal femur visualized by double calcein labels. Scale bars, 30 μm (the upper panel). Analysis of dynamic bone histomorphometric parameters of Tb.BFR/BS and Tb.MAR at the distal femur for the OI mice (the lower panel). BS, baseline; Veh, vehicle; OC-50q2wk, 50 mg/kg of Apc001OC conjugate q2wk; mab-25opw, 25 mg/kg romosozumab opw. Data are expressed as mean ± standard deviation followed by one-way ANOVA with Tukey’s *post hoc* test, n = 6 per group. ∗p < 0.05, ∗∗p < 0.01, ∗∗∗p < 0.005, ∗∗∗∗p < 0.0001.

To evaluate the effect of Apc001OC on bone mass and bone microarchitecture in OI mice, 6-week-old *Col1a2*^*+/G610C*^ mice (OI) were subcutaneously administered with 25 mg/kg of Apc001OC conjugate, Apc001HC conjugate, Apc001DA conjugate, scrambledApc001OC (scApc001OC) conjugate, Apc001PE conjugate, or non-conjugated Apc001 once every 2 weeks (q2wk) for a 12-week period.^12^^,^^33^ Micro-computed tomography (micro-CT) was utilized for the measurement of trabecular bone (below the growth plate) at the metaphysis of the distal femur in *Col1a2*^*+/G610C*^ mice. Before treatment, trabecular volume per total volume (Tb.BV/TV), trabecular volumetric mineral density (Tb.vBMD), and trabecular number (Tb.N) at the above site were significantly lower in the OI-baseline group in comparison with the wild-type (WT) baseline (group (Figure 6). It indicated substantially lower bone mass and worse bone microarchitecture for the trabecular bone in distal femur of *Col1a2*^*+/G610C*^ mice compared to WT mice. The micro-CT analysis showed that the Apc001OC group had significantly higher Tb.vBMD (+73.8%, p < 0.01), Tb.BV/TV (+128%, p < 0.01), and Tb.N (+70.6%, p < 0.01) compared to the OI-Veh group. However, our data demonstrated no differences in the levels of Tb.vBMD, Tb.BV/TV, and Tb.N among the OI-Veh, Apc001, and scApc001OC groups (Figure 5A). The levels of Tb.vBMD, Tb.BV/TV, and Tb.N in Apc001OC group were significantly higher than those in the Apc001PE group. More importantly, the levels of the above parameters in the Apc001OC group were even higher than the addition of those in Apc001HC group and Apc001DA group.

To examine the effect of Apc001OC on bone formation in OI mice, bone histomorphometric analysis was used for measurement of trabecular bone (below the growth plate) at the distal femur. Before treatment, trabecular bone formation rate/bone surface (Tb.BFR/BS) and trabecular mineral apposition rate (Tb.MAR) at the distal femur were significantly lower in the OI-baseline group compared to WT-baseline group, indicating substantially lower bone formation for trabecular bone of *Col1a2*^*+/G610C*^ mice when compared to WT mice. The bone histomorphometric analysis showed that the Apc001OC group had significantly higher BFR/BS (+208.8%, p < 0.0001) and MAR (+140.5%, p < 0.0001) compared to the OI-Veh group (Figure 5B). The Apc001HC group also had higher BFR/BS (+104.4%, p < 0.0001) and MAR (+87.9%, p < 0.005) compared to the OI-Veh group (Figure 5B). However, our data demonstrated no differences in the levels of BFR/BS and MAR among the OI-Veh, Apc001, and scApc001OC groups (Figure 5B). The levels of BFR/BS and MAR in the Apc001OC group were significantly higher than those in Apc001PE group. More importantly, the levels of the above parameters in the Apc001OC group were even higher than the addition of those in Apc001HC group and Apc001DA group.

Taken together, HC and DA could synergistically facilitate the conjugated Apc001 promoting bone formation, increasing bone mass (Tb.vBMD) and improving bone microarchitecture integrity (Tb.BV/TV, Tb.N) in *Col1a2*^*+/G610C*^ mice.

### The HC-DA combination effect on facilitating the conjugated Apc001 promoting bone formation, increasing bone mass, and improving bone microarchitecture integrity was reduced or abolished when it was pre-saturated with our designed blocking peptide sequences in *Col1a2*^*+/G610C*^ mice

From the above binding validation data, peptide A, peptide B, and peptide C could block the binding of HC, DA, and HC-DA combination to HSA, respectively. To investigate the role of HC-DA combination effect in facilitating the conjugated Apc001 promoting bone formation, increasing bone mass, and improving bone microarchitecture integrity, *Col1a2*^*+/G610C*^ mice were subcutaneously administered with Apc001OC, peptide A/B/C@Apc001OC, or blocking peptides alone, respectively. The trabecular bone in distal femur was examined by micro-CT and bone histomorphometric analysis, respectively. The micro-CT analysis showed that the Apc001OC group had significantly higher Tb.vBMD (+73.8%, p < 0.01), Tb.BV/TV (+128%, p < 0.01), and Tb.N (+70.6%, p < 0.01) compared to the OI-Veh group (Figure 5A). However, our data demonstrated no differences in the above parameters between the OI-Veh group and the other groups (Figures 5A and S10A) (p > 0.05). Our above data indicated that the HC-DA combination effect on facilitating the conjugated Apc001 increasing bone mass and improving bone microarchitecture integrity was reduced when it was pre-saturated with either peptide A or peptide B. Importantly, the HC-DA combination effect on facilitating the conjugated Apc001 increasing bone mass and improving bone microarchitecture integrity was abolished when it was pre-saturated with peptide C, indicating the important role of HC-DA combination effect on facilitating the conjugated Apc001 promoting increasing bone mass and improving bone microarchitecture integrity in OI mice. The bone histomorphometric analysis showed that the Apc001OC group had significantly higher BFR/BS (+208.8%, p < 0.0001) and MAR (+140.5%, p < 0.0001) compared to the OI-Veh group (Figure 5B). The peptide B@Apc001OC group also had higher BFR/BS (+96.7%, p < 0.0001) and MAR (+81.6%, p < 0.0001) compared to the OI-Veh group (Figure 5B). However, our data demonstrated no differences in the above parameters between the OI-Veh group and the other groups (Figures 5B and S10B) (p > 0.05). Our data demonstrated that the HC-DA combination effect on facilitating the conjugated Apc001 promoting bone formation was reduced when it was pre-saturated with either peptide A or peptide B (from +208.8% to +96.7% for BFR/BS, from +140.5% to +81.6% for MAR). Importantly, the HC-DA combination effect on facilitating the conjugated Apc001 promoting bone formation was abolished when it was pre-saturated with peptide C, indicating the important role of HC-DA combination effect on facilitating the conjugated Apc001 promoting bone formation in OI mice.

### The HC-DA interaction effect on facilitating the conjugated Apc001 promoting bone formation, increasing bone mass, and improving bone microarchitecture integrity was abolished when it was pre-saturated with peptide D in *Col1a2*^*+/G610C*^ mice

From the above binding validation data, the peptide D could block the binding of HC-DA interaction to HSA. To investigate the role of HC-DA interaction effect in facilitating the conjugated Apc001 promoting bone formation, increasing bone mass, and improving bone microarchitecture integrity, *Col1a2*^*+/G610C*^ mice were subcutaneously administered with Apc001OC, peptide D@Apc001OC, or peptide D, respectively. The trabecular bone in distal femur was examined by micro-CT and bone histomorphometric analysis, respectively. The micro-CT analysis showed that the Apc001OC group had significantly higher Tb.vBMD (+73.8%, p < 0.01), Tb.BV/TV (+128%, p < 0.01), and Tb.N (+70.6%, p < 0.01) compared to the OI-Veh group. The peptide D@ Apc001OC group also had notably higher Tb.vBMD (+54.2%, p < 0.01), Tb.BV/TV (+88.2%, p < 0.01), and Tb.N (+53%, p < 0.01) compared to the OI-Veh group (Figure 5A). Our data demonstrated that the HC-DA interaction effect on facilitating the conjugated Apc001 increasing bone mass and improving bone microarchitecture integrity was abolished when it was pre-saturated with peptide D (from +73.8% to +54.2% for Tb.vBMD, from +128% to +99.5% for Tb.BV/TV, from +70.6% to +47.3% for Tb.N), indicating the important role of HC-DA interaction effect on facilitating the conjugated Apc001 increasing bone mass and improving bone microarchitecture integrity in OI mice. The bone histomorphometric analysis showed that the Apc001OC group had significantly higher BFR/BS (+208.8%, p < 0.0001) and MAR (+140.5%, p < 0.0001) compared to the OI-Veh group. The peptide D@Apc001OC group also had notably higher BFR/BS (+124.2%, p < 0.0001) and MAR (+104%, p < 0.0001) compared to the OI-Veh group (Figure 5B). Our data demonstrated that the HC-DA interaction effect on facilitating the conjugated Apc001 promoting bone formation was abolished when it was pre-saturated with peptide D (from +208.8% to +124.2% for BFR/BS, from +140.5% to +104% for MAR), indicating the important role of HC-DA interaction effect on facilitating the conjugated Apc001 promoting bone formation in OI mice.

### Apc001OC with 50 mg/kg q2wk, showed comparable effect on promoting bone formation, increasing bone mass, and improving bone microarchitecture integrity at distal femur in *Col1a2*^*+/G610C*^ mice when compared to the marketed sclerostin antibody with 25 mg/kg once weekly

To further compare the bone anabolic potential of Apc001OC conjugate and the marketed sclerostin antibody (romosozumab), *Col1a2*^*+/G610C*^ mice were subcutaneously administered with either 50 mg/kg Apc001OC conjugate q2wk or 25 mg/kg romosozumab once weekly (opw) for 12 weeks, respectively. The trabecular bone in distal femur was examined by micro-CT and bone histomorphometric analysis, respectively. The micro-CT analysis showed that 50OC-q2wk group had significantly higher Tb.vBMD (+122%, p < 0.01), Tb. BV/TV (+217%, p < 0.0001), and Tb.N (+118%, p < 0.0001) compared to the OI-Veh group (Figure 6A). The 25mab-opw group also had significantly higher Tb.vBMD (+100%, p < 0.05), Tb.BV/TV (+176%, p < 0.01), and Tb.N (+83%, p < 0.01) compared to the OI-Veh group (Figure 6A). The bone histomorphometric analysis showed that the 50OC-q2wk group had significantly higher BFR/BS (+302.2%, p < 0.0001) and MAR (+188.2%, p < 0.0001) compared to the OI-Veh group (Figure 6B). The 25mab-opw group also had significantly higher BFR/BS (+334.4%, p < 0.0001) and MAR (+163.2%, p < 0.0001) compared to the OI-Veh group (Figure 6B). Notably, the Apc001OC group had comparable Tb.BV/TV, Tb.vBMD, Tb.N, BFR/BS, and MAR compared to the romosozumab group. Our above data indicated that Apc001OC with 50 mg/kg q2wk showed comparable effect on promoting trabecular bone formation, increasing trabecular bone mass (Tb.vBMD), and improving trabecular microarchitecture (Tb.BV/TV, Tb.N) at distal femur in *Col1a2*^*+/G610C*^ mice when compared to sclerostin antibody with 25 mg/kg opw.

## Discussion

### Design and validation of HC and DA as two candidate LMWCAs with high binding affinity to HSA

The SPR result demonstrated that HC exhibited significant high binding affinity to HSA, indicating that the SPR data validated the prediction data. The coumarin structure on HC could contribute to its high binding affinity to HSA. Importantly, the anticoagulant study demonstrated that HC had no anticoagulant effects compared to warfarin. Prior studies have implicated that the benzylacetone moiety is essential for the anticoagulant effect of warfarin.^26^ Thus, the abolished anticoagulant effect of HC could be attributed to the absence of this benzylacetone group. Our data supported that HC could be a suitable candidate LMWCA rather than warfarin.

The SPR result demonstrated that DA possessed a low binding affinity to FABP but high binding affinity to HSA, indicating that the SPR data validated the prediction data. The length of FA chains is pivotal in the binding of FAs to FABP.^32^ Consistently, the above data regarding the low binding affinity of DA to FABP but high binding affinity of DA to HSA could be explained by the fatty chain of DA being shorter than OA. Consequently, our data supported that DA could be a suitable candidate LMWCA rather than OA.

### A synergetic interaction was predicted between HC and DA on binding to HSA by the molecule-protein computational analysis and experimentally validated by the SPR assay

According to the molecule-protein computational analysis data, there were more predicted binding sites (K191, K195, R253, R238, and H238) of HSA to HC with the presence of DA near the binding pocket of HC to HSA than those (Y146 and R253) of HSA to HC without the presence of DA. This observation is consistent with SPR findings, where the Apc001OC conjugate exhibited markedly enhanced binding affinity to HSA than either Apc001HC or Apc001DA. The synergetic interaction between HC and DA could be explained by the FA structure on DA potentially modulating the conformation of the HC binding pocket on HSA, facilitating the engagement of more amino acid residues, in which the binding affinity of HC to HSA was dramatically enhanced.^29^ The above data indicated that both HC and DA could synergistically facilitate the binding affinity of the conjugated Apc001 to HSA.

### HC and DA could synergistically facilitate prolonging the half-life of the conjugated Apc001 and promoting its bone anabolic potential in OI mice via their synergistic binding to HSA

Apc001OC demonstrated longer half-life and enhanced bone anabolic potential than either Apc001HC or Apc001DA at the same dosage and dosing interval. Mechanistically, the prolonged half-life and enhanced bone anabolic potential of Apc001OC was reduced or abolished when it was pre-saturated with our designed blocking peptides. Logically, the longer half-life of Apc001OC could be explained by HC and DA synergistically binding to HSA and consequently exhibiting dramatically stronger binding affinity to HSA than either Apc001HC or Apc001DA. Moreover, the enhanced bone anabolic potential of Apc001OC could be further explained by the half-life of Apc001OC (t_1/2_ = 289.94 h) being longer than either Apc001HC (t_1/2_ = 162.93 h) or Apc001DA (t_1/2_ = 98.81 h). Together, the above data indicated that HC and DA could synergistically facilitate prolonging the half-life of the conjugated Apc001 and promoting its bone anabolic potential in OI mice via their synergistic binding to HSA.

### Apc001OC with 50 mg/kg q2wk showed comparable bone anabolic potential in OI mice when compared to the marketed sclerostin antibody with 25 mg/kg opw

It has been reported that the marketed sclerostin antibody (romosozumab) was proved to improve skeletal parameters in OI mice.^40^ In this study, Apc001OC with 50 mg/kg q2wk, showed comparable effect on promoting bone formation, increasing trabecular bone mass, and improving trabecular microarchitecture at distal femur in OI mice when compared to romosozumab with 25 mg/kg opw. Although Apc001OC was administered less frequently than romosozumab, the therapeutic effect of both drugs was comparable. This could be explained by that the half-life of Apc001OC was four times longer than romosozumab, which allows Apc001OC to achieve comparable therapeutic effect with a lower dosing frequency than romosozumab when the total dosage of both drugs is equal during the treatment. For the unmodified aptamers with short half-life, frequent administrations at a short interval could largely reduce the clinical treatment compliance of patient. Notably, this proposed bimolecular modification strategy could afford an interval of 2 weeks for the administration of the conjugated aptamer, which could potentially improve the clinical treatment compliance, especially for the treatment of chronic diseases.^19^

### Perspective

In general, this proposed bimolecular modification strategy could greatly extend the half-life of the conjugated sclerostin aptamer (up to 12 days) compared to that of PEGylation (only 2.7 days), which could potentially improve the clinical treatment compliance.^12^^,^^33^ Concurrently, the bimolecular modification strategy could remarkably increase the proportion of aptamer within the aptamer conjugate (up to 95%) compared to that of PEGylation (low to 25%), which increases the subcutaneous dosage for the aptamer moiety at a fixed subcutaneous administration volume. Accordingly, the synergistic effect between HC and DA may pave the way for development of all long-lasting therapeutic aptamers targeting circulating proteins with increasable dosages. Although existing studies corroborate the superior efficacy of multiple FAs in enhancing the binding of warfarin to HSA compared to singular FA, the optimal amount of DA required to enhance the binding of HC to HSA remains ambiguous. Thus, the tailored combination of HC with varying DA amounts needs to be further explored for pharmacokinetics-related aptamer optimization. On the other hand, it is worth screening different combinations of HC derivatives and DA derivatives to enhance their binding affinity to HSA for pharmacokinetics-related aptamer optimization. However, combinations of HC derivatives (>10^5^) and DA derivatives (>10^5^) could be at least 10^10^, all of which cannot be experimentally implemented due to the high cost in both labor and time. Artificial intelligence could be employed to generate both HC derivatives and DA derivatives with predicted binding affinities to HSA for further predicting the most promising HC derivative/DA derivative combination.^41^ Moreover, integrating computational chemistry with structural biology is imperative for a comprehensive understanding of the Apc001OC@HSA complex.

### Conclusions

In summary, two innovative coupling agents are designed, HC and DA, which could synergistically bind to HSA with high affinities. The *in vivo* study indicated that HC and DA could synergistically facilitate prolonging the half-life of the conjugated Apc001 and promoting its pro-anabolic potential in OI mice via their synergistic binding to HSA. Importantly, Apc001OC showed dramatically longer half-life and enhanced pro-anabolic potential compared to Apc001PE. Furthermore, Apc001OC with 50 mg/kg q2wk shows comparable bone anabolic potential to the marketed sclerostin antibody with 25 mg/kg opw. The proposed innovative long-lasting modification strategy could help address the druggability challenge of aptamers with short half-life. Future studies would aim to use artificial intelligence to generate both HC derivatives and DA derivatives with predicted binding affinities to HSA for further predicting the most promising HC derivative/DA derivative combination.

## Materials and methods

### Molecular-protein docking by Schrodinger

Schrodinger_Suites_2021-2 is a suite of software for simulating small molecule and macromolecule systems. It was used to perform docking simulations of the coumarin derivatives to full-length HSA (PDB: 1AO6). Some docking modes were generated. HSA were prepared using Protein Preparation Wizard with default parameters. All small molecules were prepared using LigPrep. Additionally, the ionization of LigPrep was set to generate possible states at target pH 7.0 ± 0.5 using Epik, and the checkbox to include the original state was checked. Before running Glide Grid Generation, the grid box was set to cover the whole macromolecule. Before running Glide Ligand Docking, the precision of Glide ligand docking was set to XP (extra precision) and the checkbox of Add Epik state penalties to docking score was unchecked. The output parameters of Glide ligand docking were selected as follows: the maximum number of poses per ligand written out was selected as 25 and the number of poses per ligand to include was selected as 500. All coumarin derivatives in this study were selected from the PubChem database. The output format of the coumarin derivatives was based on REST-style version of Power User Gateway (PUG) (https://pubchemdocs.ncbi.nlm.nih.gov/pug-rest), and all compounds were further refined offline. Due to the size of the binding pocket of the receptor and Lipinski rule of five, molecules with molecular weight higher than 550 were removed. The final screening library contained 14,991 coumarin derivatives without a benzylacetone group. AutoDock Vina was used for the virtual screening. The top eight conformations with comparably low binding energy to HSA were chosen for subsequent extra precision docking by Schrodinger. The top eight predicted coumarin derivatives with comparably high predicted binding affinity to HSA are shown in Table S2. Among the eight coumarin analogues, 4-hydroxycoumarin possessed the highest predicted binding affinity (energy, −6.91 kcal/mol; rank, 1) to HSA.

### Molecular-protein docking by AutoDock

AutoDockTools 1.5.6 is a suite of software for simulating small-molecule and macromolecule systems. It was used to perform docking simulation of the truncated OA derivatives to full-length HSA (PDB: 1AO6) and FABP (PDB: 1G5W), respectively. Some docking modes were generated. Both HSA and FABP should delete water and add hydrogens before setting as macromolecules. All small molecules should add hydrogens before setting as ligand. Additionally, the number of rotatable bonds of ligands was selected by software automatically. Before running autogrid, the grid box should be set to cover the whole macromolecule, and the ligand should be outside the grid box. Before running autodock, macromolecule should be set as a rigid receptor. Both user-specified initial position and initial relative dihedral offset (quat0) were random. Genetic algorithm parameters were selected as follows: number of GA Runs was selected 20; maximum number of evals was selected 2,500,000; maximum number of generations was selected 27,000. All the docking parameters were set to defaults, and Lamarckian Genetic Algorithm 4.2 (LGA) was selected to calculate the lowest energy of the system. The docking results are shown in Table S3. Among all the tested truncated OA derivatives, dodecanedioic acid demonstrated the lowest predicted binding affinity (energy, −0.23 kcal/mol; rank, 1) to FABP (PDB: 1G5W) but comparably high predicted binding affinity to HSA (PDB: 1AO6).

### Molecular dynamics simulation

Amber20 is a suite of biomolecular simulation programs for simulating small-molecule and macromolecule systems. It was used to perform molecular dynamics simulations of the coumarin moiety of HC to full-length HSA (PDB: 1AO6) with the presence of the FA moiety of DA. All the small molecules were prepared by the antechamber module and then checked by the parmchk2 module separately. The HSA was prepared by the pdb4amber module and the reduce module. After the preparation of small molecules and HSA, all the structures were combined and prepared using the LEaP module. Additionally, the protein force field was set as ff14SB and the water model was set as TIP3P. Then the PMEMD.CUDA module was used as the molecular dynamics engine to perform molecular dynamics simulations. Cluster analysis was performed once the molecular dynamics simulation process was completed. The binding pocket of HC was located inside the HSA structure. Both HC and DA were conjugated the Apc001 to form an Apc001OC; therefore, the binding sites between DA and HSA should be near the HC binding pocket, which was within the distance of 30 Å. As a result, three binding pockets (Z1, Z2, Z3; Figure S19) within a distance of 30 Å from the HC binding pocket were selected. First, we conducted molecular dynamics simulations with a short timescale (13 ns) to predict the binding modes between HC and HSA, with or without the presence of DA in the three binding pockets. The results are shown in Figure S20. With the presence of DA in pocket Z3, HC possessed the highest binding affinity to HSA. There were four predicted binding sites between HC and HSA: Y146, K195, H238, and R253. In order to obtain more reliable results, we further conducted a molecular dynamics simulation with a longer timescale (30 ns) for precise prediction of binding affinity of HC to HSA, with the presence of DA in pocket Z3. The predicted results are summarized in Figure 1. Without the presence of DA, there were two predicted binding sites between HC and HSA: Y146 and R253. Notably, with the presence of DA in pocket Z3, there were five predicted binding sites between HC and HSA: K191, K195, R218, H238, and R253. Also, R253 was the only unaltered predicted binding site of HC to HSA, with or without the presence of DA in pocket Z3.

### Preparation of DA

In a flame-dried 100-mL flask, dodecanedioic acid (2.30 g, 10.0 mmol, 1.0 equiv) was dissolved in 30 mL of anhydrous tetrahydrofuran (THF) by stirring at 0°C. DCC (2.68 g, 13.0 mmol, 1.3 equiv) was added slowly to the mixture. After 10 min, the solution of 1-hydroxypyrrolidine-2, 5-dione (1.15 g, 10.0 mmol, 1.0 equiv) in 5.0 mL of anhydrous THF was added to the mixture at the same temperature. After 20 min, 4-DMAP (20.0 mg) was added to the mixture. The resulting mixture was warmed up slowly to room temperature and stirred overnight. The solid was filtered off and washed with THF. The filtrate was concentrated *in vacuo* and products were purified by column chromatography (eluent: dichloromethane/MeOH = 40:1–20:1, v/v) to afford the pure product DA (2.05 g, 62.6% yield), a white solid. High resolution electrospray ionization mass spectrometry (ESI-HRMS) calculated for C_16_H_26_NO_6_^+^, 328.1755 [M + H]+; found 328.1754 [M + H]+.

### Preparation of HC

In a flame-dried 100-mL flask, 3-(2,5-dioxo-2,5-dihydro-1H-pyrrol-1-yl)propanoic acid (1.01 g, 6.0 mmol, 1.0 equiv), HBTU (2.73 g, 7.2 mmol, 1.2 equiv), and DIPEA (1.2 mL, 7.2 mmol, 1.2 equiv) were dissolved in 30 mL of DMF by stirring at room temperature. After 20 min, the solution of 6-amino-4-hydroxy-2H-chromen-2-one (1.06 g, 6.0 mmol, 1.0 equiv) in 1.0 mL of DMF was added to the mixture. The resulting mixture was stirred overnight. The mixture was diluted into 50 mL of water and then extracted with ethyl acetate (3 × 30 mL), and the extracted liquid with ethyl acetate was dried over Na_2_SO_4_. The filtrate was concentrated *in vacuo* and products were purified by column chromatography (eluent: DCM/MeOH = 40:1–20:1, v/v) to afford the pure product HC (1.5 g, 76% yield), a yellow solid. ESI-HRMS calculated for C_16_H_13_N_2_O_6_^+^, 300.0608 [M + H]+; found 300.0613 [M + H]+.

### NMR analysis of both DA and HC

NMR spectroscopy was used to determine the structures of small molecules. All NMR spectra were recorded on a Bruker spectrometer at 400 MHz (^1^H NMR), 101 or 126 MHz (^13^ C NMR). Chemical shifts (δ value) were reported in ppm with the solvent signals as reference (d-DMSO as solvent) and coupling constants (*J*) are given in Hertz (Hz). The peak information was described as br, broad singlet; s, singlet; d, doublet; t, triplet; q, quartet; m, multiplet; comp, composite of magnetically non-equivalent protons, downfield from internal tetramethylsilane (TMS). There were 25 protons in ^1^H NMR spectra and 16 carbons in ^13^C NMR spectra, respectively, which were matched the actual number of either protons or carbons of DA (C_16_H_25_NO_6_). ^1^H NMR (400 MHz, DMSO) δ 11.95 (s, 1H), 2.81 (s, 4H), 2.65 (t, *J* = 7.2 Hz, 2H), 2.18 (t, *J* = 7.4 Hz, 2H), 1.66–1.57 (m, 2H), 1.53–1.45 (m, 2H), and 1.25 (s, 12H) (Figure S11A). ^13^C NMR (101 MHz, DMSO) δ 174.5, 170.2, 168.9, 33.6, 30.2, 28.8, 28.8, 28.7, 28.5, 28.5, 28.0, 25.4, 24.5, and 24.3 (Figure S11B). There were 12 protons in ^1^H NMR spectra and 16 carbons in ^13^C NMR spectra, respectively, which were matched the actual number of either protons or carbons of HC (C_16_H_12_N_2_O_6_). ^1^H NMR (400 MHz, DMSO) δ 12.53 (s, 1H), 10.23 (s, 1H), 8.16 (d, *J* = 2.5 Hz, 1H), 7.66 (dd, *J* = 8.9, 2.6 Hz, 1H), 7.32 (d, *J* = 8.9 Hz, 1H), 7.03 (s, 2H), 5.60 (s, 1H), 3.73 (t, *J* = 7.0 Hz, 2H), and 2.61 (t, *J* = 7.0 Hz, 2H) (Figure S12A). ^13^C NMR (101 MHz, DMSO) δ 170.8, 168.6, 165.5, 161.9, 149.3, 135.0, 134.6, 123.9, 116.6, 115.7, 112.7, 91.2, 35.1, and 33.8 (Figure S12B).

### Preparation of the Apc001-SH-NH_2_

The Apc001-SH-NH_2_ (sequence: NH_2_-C4-(SH-C6C1)C2- C(OMe)G(OMe)G(OMe)G(OMe)GTGTGGGTTCGTCGTTAGCTTGATTTGGCAGCU(OMe)G(OMe)C(OMe)C(OMe)-idT) was synthesized on a 32-μmol scale on an ÄKTA oligopilot plus 100 standard DNA/RNA synthesizer using commercially available 3′-IDT CPG, 5′-O-DMT-2′-deoxynucleoside (A_Bz_, C_Ac_, G_iBu_, and T) phosphoramidite monomers, 5′-O-DMT-2′-O-methyl nucleoside (A_Bz_, C_Ac_, G_iBu_ and U) phosphoramidite monomers, amino-modifier C7 CE phosphoramidite monomer, and thiol-modified C6 S-S phosphoramidite monomer. All oligonucleotides were synthesized in DMT-OFF mode. After completion of the synthesis, the solid support was suspended in 28% ammonium hydroxide solution and heated at 60°C for 4 h to release the product from the support and to complete the removal of all protecting groups. The solid support was filtered, and the filtrate was desalted/buffer exchanged into ddH_2_O and lyophilized to dryness. The mixture was then dissolved in 10 mL of pH = 8.4 ddH_2_O by stirring at room temperature and 100 mM dithiothreitol (DTT) solution was added to the mixture. The resulting mixture was stirred for 2 h. After the reaction was completed, the resulting mixture was lyophilized to dryness.

### Preparation of Apc001-SH

The Apc001-SH (sequence: SH-C6-C(OMe)G(OMe)G(OMe)G(OMe)GTGTGGGTTCGTCGTTAGCTTGATTTGGCAGCU(OMe)G(OMe)C(OMe)C(OMe)-idT) was synthesized on a 32-μmol scale on an ÄKTA oligopilot plus 100 standard DNA/RNA synthesizer using commercially available 3′-IDT CPG, 5′-O-DMT-2′-deoxynucleoside (A_Bz_, C_Ac_, G_iBu_, and T) phosphoramidite monomers, 5′-O-DMT-2′-O-methyl nucleoside (A_Bz_, C_Ac_, G_iBu_ and U) phosphoramidite monomers, and thiol-modified C6 S-S phosphoramidite monomer. All oligonucleotides were synthesized in DMT-OFF mode. After completion of the synthesis, the solid support was suspended in 28% ammonium hydroxide solution and heated at 60°C for 4 h to release the product from the support and to complete the removal of all protecting groups. The solid support was filtered, and the filtrate was desalted/buffer exchanged into ddH_2_O and lyophilized to dryness. The mixture was then dissolved in 10 mL of pH = 8.4 ddH_2_O by stirring at room temperature and 100 mM DTT solution was added to the mixture. The resulting mixture was stirred for 2 h. After reaction was completed, the resulting mixture was lyophilized to dryness.

### Preparation of the Apc001-NH_2_

The Apc001-NH_2_ (sequence: NH_2_-C6-C(OMe)G(OMe)G(OMe)G(OMe)GTGTGGGTTCGTCGTTAGCTTGATTTGGCAGCU(OMe)G(OMe)C(OMe)C(OMe)-idT) was synthesized on a 32-μmol scale on an ÄKTA oligopilot plus 100 standard DNA/RNA synthesizer using commercially available 3′-IDT CPG, 5′-O-DMT-2′-deoxynucleoside (A_Bz_, C_Ac_, G_iBu_, and T) phosphoramidite monomers, 5′-O-DMT-2′-O-methyl nucleoside (A_Bz_, C_Ac_, G_iBu_ and U) phosphoramidite monomers, and amino-modifier C6 CE phosphoramidite monomer. All oligonucleotides were synthesized in DMT-OFF mode. After completion of the synthesis, the solid support was suspended in 28% ammonium hydroxide solution and heated at 60°C for 4 h to release the product from the support and to complete the removal of all protecting groups. The solid support was filtered, and the filtrate was desalted/buffer exchanged into ddH_2_O and lyophilized to dryness.

### Purification of the modified Apc001

The crude modified Apc001 was dissolved in 40 mL of ddH_2_O, and then the mixture was filtered with 0.45-μm polytetrafluoroethylene (PTFE) membrane to obtain clear filtrate. The crude product was purified by preparative liquid chromatography with the Autotide 100 Oligo Purification System to obtain the modified Apc001 (condition: column, Autotide-RP-20 C18, 250 × 22 mm, 1 mm; 26°C; a flow rate of 6 mL min ^−1^; a linear gradient of 5–35% v/v solvent B in 30 min, A was 0.05 M triethylamine acetate [TEAA] solution, B was HPLC grade acetonitrile).

### Preparation of the Apc001OC conjugate

In a flame-dried 100-mL flask, HC (240 mg, 100 equiv) was dissolved in 4 mL of DMF by stirring at room temperature. Then the solution of 100 mg of Apc001-SH-NH_2_ in 6 mL of 1× PBS solution (pH = 6.71) was added to the mixture. The resulting mixture was stirred overnight. The solution of NaHCO_3_ (150 mg) in 3 mL of ddH_2_O was added to the mixture. Then the solution of DA (250 mg, 100 equiv) in 2 mL of DMF was added to the mixture. The resulting mixture was stirred overnight again. After reaction was completed, the mixture was concentrated *in vacuo* to obtain the crude Apc001OC conjugate.

### Preparation of the Apc001HC conjugate

In a flame-dried 100-mL flask, HC (240 mg, 100 equiv) was dissolved in 4 mL of DMF by stirring at room temperature. Then the solution of 100 mg of Apc001-SH-NH_2_ in 6 mL of 1× PBS solution (pH = 6.71) was added to the mixture. The resulting mixture was stirred overnight. After reaction was completed, the mixture was concentrated *in vacuo* to obtain the crude Apc001HC conjugate.

### Preparation of the Apc001DA conjugate

In a flame-dried 100-mL flask, NaHCO_3_ (150 mg) was dissolved in 4 mL of ddH_2_O by stirring at room temperature. Then the solution of DA (250 mg, 100 equiv) in 2 mL of DMF was added to the mixture, and 100 mg of Apc001-NH_2_ in 6 mL of ddH_2_O was subsequently added. The resulting mixture was stirred overnight. After reaction was completed, the mixture was concentrated *in vacuo* to obtain the crude Apc001DA conjugate.

### Purification of theApc001OC/HC/DA conjugate

The crude Apc001OC/HC/DA conjugate was dissolved in 20 mL of ddH_2_O, and then the mixture was filtered with 0.45-μm PTFE membrane to obtain clear filtrate.^42^^,^^43^ The crude product was purified by preparative liquid chromatography with the Autotide 100 Oligo Purification System to obtain the pure Apc001OC/HC/DA conjugate (condition: column, Autotide-RP-20 C18, 250 × 22 mm, 1 mm; 26°C; a flow rate of 6 mL min ^−1^; a linear gradient of 5–35% v/v solvent B in 40 min, A was 0.05 M TEAA solution, B was HPLC grade acetonitrile).

### Mass spectrometric analysis

The mass of aptamers and aptamer conjugates was determined by an ultra-HPLC coupled with quadrupole time-of-flight mass spectrometry (UHPLC-ESI-Q-TOF-MS) in negative ion mode on a single quadrupole liquid chromatograph mass spectrometer (Xevo G2-XS QTof, Waters). The mass of purified Apc001-SH-NH_2_ was confirmed by ESI-Q-TOF-MS. The actual measured weight (13341.0654) matched the calculated weight (13,348.7711) (Figure S13). The mass of purified Apc001-SH was confirmed by ESI-Q-TOF-MS. The actual measured weight (13,139.5156) matched the calculated weight (13,139.5811) (Figure S14). The mass of purified Apc001-NH_2_ was confirmed by ESI-Q-TOF-MS. The actual measured weight (13,117.9912) matched the calculated weight (13,122.5415) (Figure S15). The mass of purified Apc001OC conjugate was confirmed by ESI-Q-TOF-MS. The actual measured weight (13,882.9863) matched the calculated weight (13,889.3463) (Figure S16). The mass of purified Apc001HC conjugate was confirmed by ESI-Q-TOF-MS. The actual measured weight (13,452.0605) matched the calculated weight (13,467.8685) (Figure S17). The mass of purified Apc001DA conjugate was confirmed by ESI-Q-TOF-MS. The actual measured weight (13,329.1309) matched the calculated weight (13,334.8350) (Figure S18).

### SPR analysis

Every protein (or peptide) was immobilized on the CM5 chip (GE) by the method of amine coupling. Conditions for immobilization of protein (or peptide) contained several parameters as follows: surface activation and ligand attachment were performed at 25°C; specified contact time was 420 s; the concentration of protein or peptide was 50–80 μg/mL (every protein or peptide was dissolved in 10 mM acetate, pH = 4.5 or 5.0, according to the results of immobilization pH scouting); the aqueous buffer of aptamer conjugate ligands was 1× PBS; and the aqueous buffer of small-molecule ligands was 1× PBS with 5% DMSO. Other chemicals required for immobilization included 50 mM NaOH solution, ethanolamine, EDC, and NHS. The regeneration scouting contained several parameters as follows: number of regenerations was one; the Prime before run option was selected; contact time was 60 s; stabilization period was 5 s; number of conditions was 3 s; and number of cycles for each condition was five. The regeneration solution for regenerating protein (or peptide) was 10 mM glycine-HCl solution (pH = 3.0 or 3.5). The mode of multi-cycle kinetics with a 1:1 fit was selected to perform experiments. Different concentrations of ligands were used for analyzing the binding affinities to proteins. The buffer and flow cell-interaction effects have been subtracted from the binding curves.

### The design of blocking peptides

Based on the calculated binding sites of HSA to the coumarin moiety of HC, it was predicted that Y146 and R253 on HSA were involved. Then, we designed a peptide sequence (peptide A) including the above binding sites and verified its binding affinity to HC by SPR analysis (Figure S4A). Based on the calculated binding sites of HSA to the FA moiety of DA, it was predicted that K16, K132, and K158 on HSA were involved. Then, we designed a peptide sequence (peptide B) including the above binding sites and verified its binding affinity to DA by SPR analysis (Figure S4B). Based on the above prediction, a peptide sequence including the above binding sites that could bind to HC-DA combination was designed (peptide C). Then, we verified its binding affinity to both HC and DA by SPR analysis (Figure S4C). Based on the calculated binding sites of HSA to HC in the presence and absence of DA (Figure S5A), it was predicted that Y146 and R253 on HSA were involved in the absence of DA and K191, K195, R253, R238, and H238 on HSA were involved in the presence of DA. Interestingly, K191, K195, R238, and H238 were the newly participated binding sites in the presence of DA (Figure S5B). Then, we designed a peptide sequence (peptide D) including the above newly participated binding sites and verified its binding affinity to HC by SPR analysis (Figure S5C).^6^^,^^7^ All the peptide sequences were purchased from GL Biochem (Shanghai).

### Pharmacokinetics experiment

The pharmacokinetics study of the Apc001OC conjugate (6.25 mg/kg), the Apc001DA conjugate (6.25 mg/kg), the Apc001HC conjugate (6.25 mg/kg), peptide A@Apc001OC (6.25 mg/kg peptide A + 6.25 mg/kg Apc001OC), peptide B@Apc001OC (6.25 mg/kg peptide A + 6.25 mg/kg Apc001OC), peptide C@Apc001OC (6.25 mg/kg peptide A + 6.25 mg/kg Apc001OC), and peptide D@Apc001OC (6.25 mg/kg peptide A + 6.25 mg/kg Apc001OC) was performed in 6-month-old male Sprague-Dawley rats. The rats were fed *ad libitum* with a standard laboratory diet and housed under controlled conditions (12 h light cycle, 20°C). The rats were treated with the three compounds at 6.25 mg/kg by a single subcutaneous injection. Blood samples were collected at different time points (0, 1, 2, 4, 8, 12, 24, 36, 48, 60, 72, 84, 96, 108, 120, 144, 168, 192, 216, 240, 264, 312, 360, 408, 456, and 504 h) from rats (n = 3) and plasma was isolated. After being treated with methanol, the remaining compounds in the plasma were quantified by molecular beacon (MB). The pharmacokinetics profiles of the three compounds were analyzed by software DAS 2.0.

### Blood sampling

After subcutaneous injection of the Apc001OC conjugate, the Apc001DA conjugate, the Apc001HC conjugate, peptide A@Apc001OC, peptide B@Apc001OC, peptide C@Apc001OC, and peptide D@Apc001OC, 500 μL of blood was taken via orbital vein from each rat at different time points and was collected into tubes containing sodium heparin as an anticoagulant (0.9-mL vacutainers, BD Biosciences) and then were put into wet ice immediately.^44^

### Plasma isolation

Plasma was isolated by centrifugation at 6,000 × *g* for 10 min at 4°C within 1 h after collection and stored at −80°C until analysis. After allowing each sample to thaw, the plasma was mixed and an aliquot (100 μL) transferred to a polypropylene tube. Methanol (1.4 mL) was added to each sample and, after mixing, the plasma protein was removed by centrifugation at 14,000 × *g* for 10 min. An aliquot of the supernatant was transferred to a 250-μL limited-volume insert vial for Aptamer conjugate quantification. Then the supernatants were dried and reconstituted for aptamer quantification. Standards will be prepared in blank rat plasma containing sodium heparin.^45^

### Aptamer conjugate quantification

All the Apc001OC conjugate, the Apc001DA conjugate, and the Apc001HC conjugate could be assayed by MB technology.^46^ The MB sequence was Cy3-CCGCGCATCAAGCTAACGACGAAGCGCGG-BHQ2. The sequence of Apc001 was CmGmGmGmGTGTGGGTTCGTCGTTAGCTTGATTTGGCAGCUmGmCmCm-idT (the underlined sequence is the complementary sequence between MB and Apc001). The 96-well plate was used for loading samples. For each well, 5-μL plasma samples, 5 μM MB sequence, and 85 μL of PBS were added. Then, the reagents were mixed by gently tapping the sides of the plate. The plate was incubated and protected from light for 1 h at 37°C. The Apc001 could be detected when hybridization occurred between the complementary sequence of MB and Apc001. This caused the separation of the stem and hence of the fluorophore and the quencher. Once the fluorophore was no longer next to the quencher, illumination of the hybrid with light resulted in the fluorescent emission. The presence of the emission showed that the event of hybridization had occurred and hence the Apc001 sequence was present in the test sample. The plate was read using a fluorescence plate reader at excitation 550 nm and emission 575 nm. The concentrations of Apc001OC conjugate, the Apc001DA conjugate, and the Apc001HC conjugate were calculated according to the standard curves. Standard curves were obtained by analyzing different concentrations of Apc001 that were dissolved in plasma and processed as well as being analyzed using the same procedure.

### The pharmacokinetics analysis

The Apc001OC conjugate, the Apc001DA conjugate, and the Apc001HC concentrations versus time profile were plotted and analyzed for each rat by software DAS 2.0 (BioGuider, Shanghai, China). All reported concentrations of the Apc001OC conjugate, the Apc001DA conjugate, and the Apc001HC conjugate were based on the area under the curve (AUC). Using the data from standards, calibration curves were generated for the Apc001OC conjugate, the Apc001DA conjugate, and the Apc001HC conjugate, respectively. Half-life (t_1/2_) of the Apc001OC conjugate, the Apc001DA conjugate, and the Apc001HC conjugate were calculated according to the time it takes for the Apc001OC conjugate, the Apc001DA conjugate, and the Apc001HC conjugate to eliminate half of the maximum plasma concentration.^47^ Maximum plasma concentration (C_max_) and the time to maximum plasma concentration (T_max_) were obtained according to pharmacokinetics curves. The AUC was computed starting at the time the drug was administered and ending when the concentration in plasma was negligible.^48^^,^^49^^,^^50^ Subsequently, the dosing intervals in multiple doses of the Apc001OC conjugate was calculated according to the equation D = 1/1 − e^−^^Ke·t^ (Ke is elimination constant, Ke is ln2/T_1/2_ t is dosing interval; D is dosing ratio = loading dose/maintenance dose).^51^^,^^52^ The results are shown in Figures 4 and S7.

### Mice and genotyping

The *Col1a2*^*+/G610C*^ mice were purchased from the Laboratory Animal Services Centre (LASEC) in the Chinese University of Hong Kong (CUHK). The *Col1a2*^*+/G610C*^ were genotyped using DNA extracted from mouse tail clippings, amplified using the forward primer 5′-TCCCTGCTTGCCCTAGTCCCAAAGATCCTT-3′ and the reverse primer 5′-AAGGTATAGATCAGACAGCTGGCACATCCA-3′ to generate a 165-bp (WT), a 337-bp and a 165-bp (heterozygous), or a 337-bp (homozygous) amplicons. Genotyping was conducted by REDExtract-N-Amp Tissue PCR Kit (Sigma-Aldrich).

### An OI mouse model for examining bone

Six-week-old *Col1a2*^*+/G610C*^ mice and 6-week-old WT littermates were employed to examine the bone anabolic potential of Apc001OC in OI mice. Briefly, six 6-week-old *Col1a2*^*+/G610C*^ mice (OI-baseline) and six 6-week-old WT littermates (WT-baseline) were euthanized before treatment as baseline. Another six 6-week-old *Col1a2*^*+/G610C*^ mice (OI-age matched) and six 6-week-old WT littermates (WT-age matched) were kept untreated for 12 weeks as the age-matched groups. The remaining *Col1a2*^*+/G610C*^ mice were subcutaneously administered with Apc001OC (25 mg/kg); Apc001OC (50 mg/kg); Apc001HC (25 mg/kg); Apc001DA (25 mg/kg); peptide A@Apc001OC (25 mg/kg peptide A + 25 mg/kg Apc001OC); peptide B@Apc001OC (25 mg/kg peptide B + 25 mg/kg Apc001OC); peptide C@Apc001OC (25 mg/kg peptide C + 25 mg/kg Apc001OC); peptide D@Apc001OC (25 mg/kg peptide D + 25 mg/kg Apc001OC); or peptide A, B, C, D (25 mg/kg), q2wk for 12 weeks (n = 6 for each group) and romosozumab (25 mg/kg) opw for 12 weeks (n = 6 for each group). The administration dose of Apc001OC, Apc001HC, Apc001DA, romosozumab, and blocking peptides referred to their mass. Before euthanasia, all mice were intraperitoneally injected with two doses of fluorescent dyes at 10 and 3 days (20 mg/kg calcein). After euthanasia, the left distal femoral metaphysis was subjected to micro-CT analysis (version 6.5, vivaCT40, Scanco Medical, Bassersdorf, Switzerland) and bone histomorphometric analysis.

### Micro-CT analysis

Analysis of the trabecular bone in distal femur was performed with micro-CT (version 6.5, vivaCT40, Scanco Medical, Bassersdorf, Switzerland). Images of femur were reconstructed and calibrated at the isotropic voxel size of 12.5 and 17.5 μm (70 kVp, 114 μA, 200 ms integration time, 260 thresholds, 1,200 mg HA/cm^3^). Every measurement used the same filtering and segmentation values. Using the Scanco evaluation software, regions of interest (ROIs) were defined for trabecular parameters. For the trabecular bone, a central region was selected equivalent to 70% of the vertebral body height and extended from proximally to the end of the distal growth plate toward the vertebral body. We drew freehand the trabeculae ROI on 100 sequential slices to ensure it was within the endosteal envelope. Trabecular bone parameters, including Tb.vBMD, Tb.BV/TV, Tb.N, trabecular thickness (Tb.Th), and trabecular spacing (Tb.Sp) were calculated.^53^^,^^54^

### Bone histomorphometric analysis

After sacrificing, the left distal femoral metaphysis and the left femoral mid-shaft were fixed in 4% paraformaldehyde for 48 h; dehydrated in increased 10%, 20%, and 30% concentrations of sucrose (dilution in 1× PBS) for 24 h in each concentration; and embedded without decalcification in an optimal cutting temperature compound (Sakura Finetek, Tokyo, Japan). After embedding, the proximal region of the samples was sectioned longitudinally, and the histomorphometric analyses of trabecular bone was performed at the above four sites. The frozen tissue specimens were obtained at a thickness of 5 μm with CryoStar NX50 (Thermo Fisher Scientific, Waltham, MA, USA). The sites were consistent with the selected sites of micro-CT. Fluorescence micrographs for the bone sections were captured by a Q500MC fluorescence microscope (Leica, Bensheim, Germany). The parameters of bone dynamic histomorphometric analysis for trabecular bone and cortical bone included BFR/BS and MAR. The analysis was performed using professional histomorphometric analysis system (BIOQUANT OSTEO, Nashville, TN, USA), and the parameters were calculated and expressed according to the American Society for Bone and Mineral Research standardized nomenclature for bone histomorphometry.^55^

### Statistical analysis

All variables were expressed as mean ± standard deviation. One-way ANOVA with Tukey’s *post hoc* test was performed to determine the inter-group differences in the study variables, including for anticoagulant assay, micro-CT parameters, and bone histomorphometric parameters. All the statistical data were analyzed by GraphPad Prism (version 8; GraphPad Software, San Diego, CA, USA), and p < 0.05 was considered to be statistically significant. For the *in vivo* experiments, sample size was pre-determined by a power calculation according to our previously published protocol. The animals were grouped randomly and blindly to researchers. The animals in poor body condition were excluded.

## Data and code availability

The data used to support the findings of this study are included within the article.

## Supplemental information

Document S1. Figures S1–S21 and Tables S1–S3

Document S2. Article plus supplemental information
