# Supplementary material for: A bimolecular modification strategy for developing long-lasting bone anabolic aptamer
Source: Mol Ther Nucleic Acids. 2023 Nov 10;34:102073. doi: 10.1016/j.omtn.2023.102073 (PMC10709176; doi:10.1016/j.omtn.2023.102073)
Supplement: Document S1. Figures S1–S21 and Tables S1–S3 [file mmc1.pdf]

OMTN, Volume *34*

Supplemental information

A bimolecular modification strategy for developing long-lasting bone anabolic aptamer

Huarui Zhang, Sifan Yu, Shuaijian Ni, Amu Gubu, Yuan Ma, Yihao Zhang, Haitian Li, Yuzhe Wang, Luyao Wang, Zongkang Zhang, Yuanyuan Yu, Aiping Lyu, Baoting Zhang, and Ge Zhang

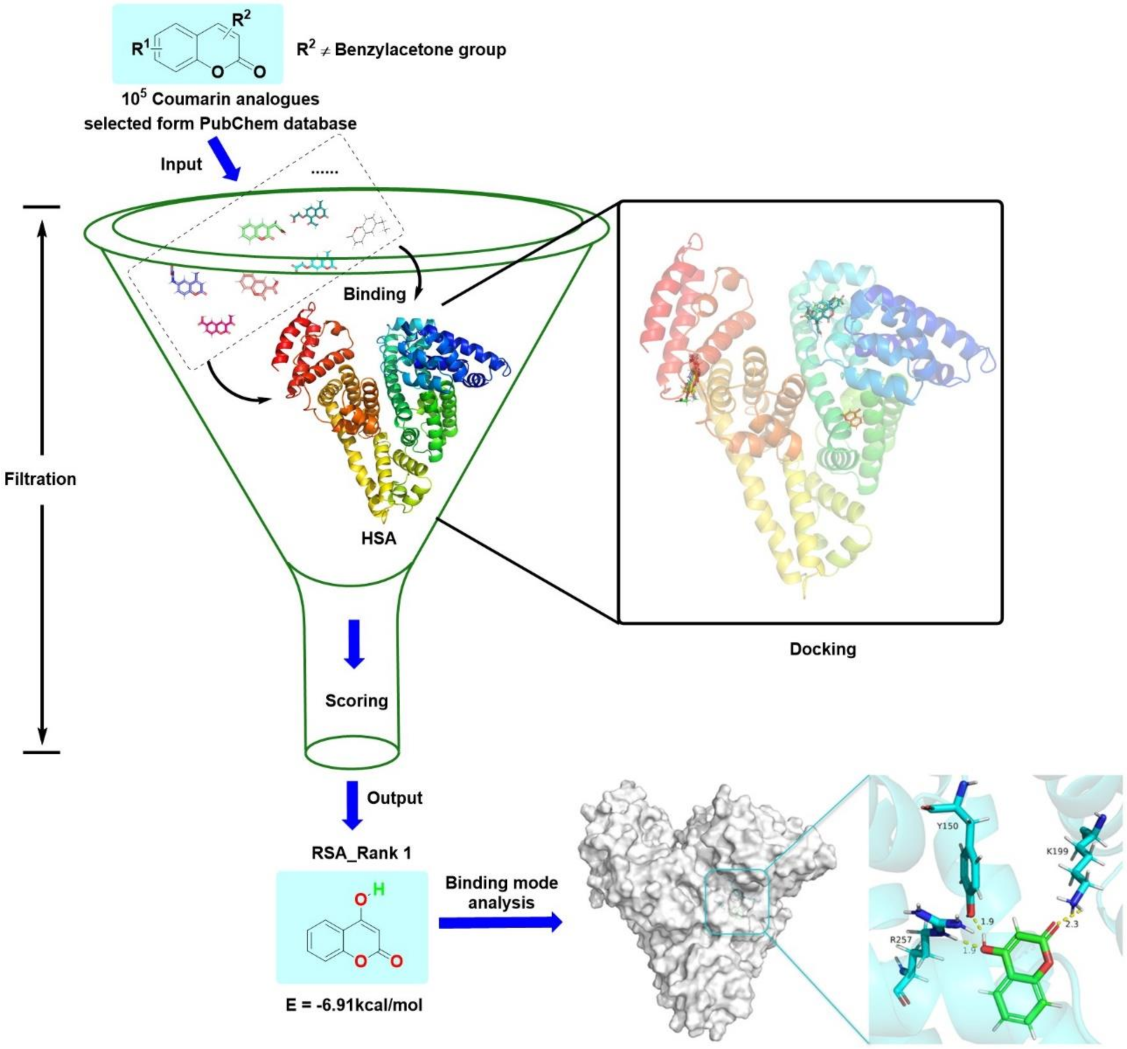

Figure S1. The schematic diagram of the high-throughput virtual screening approach to screen out a 4-hydroxycoumarin which possessed the highest predicted binding affinity from 10^5 coumarin derivatives without benzylacetone group.

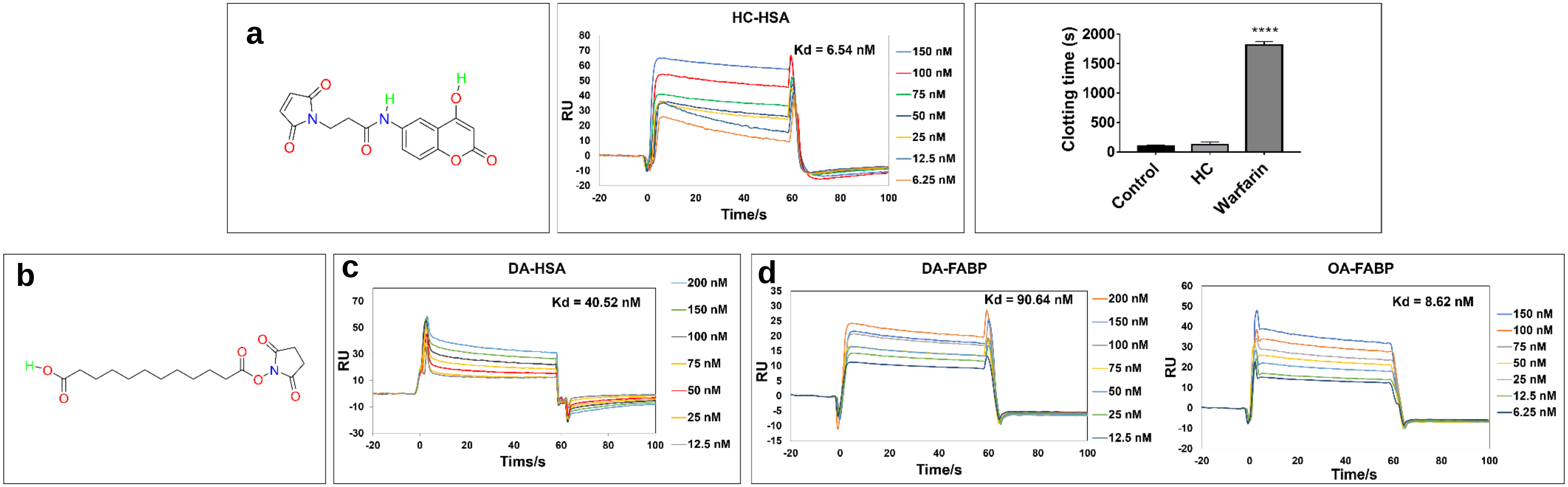

Figure S2. HC and DA could synergistically bind to human serum albumin (HSA) with high affinities. (a) Left: The structure of HC. **Middle:** The multi cycle kinetics analysis of binding affinity of HC to HSA (Kd=6.54 nM). **Right:** Warfarin markedly reduced the clotting time, whereas HC had no anticoagulant effect. **(b)** The structure of DA. **(c)** The multi cycle kinetics analysis of binding affinity of DA to HSA (Kd=40.52 nM). **(d)** DA possessed low binding affinity to FABP when compared to OA. Left: The multi cycle kinetics analysis of binding affinity of DA to fatty acid-binding proteins (FABP) (Kd=90.64 nM). Right: The multi cycle kinetics analysis of binding affinity of OA to FABP (Kd=8.62 nM). **Note:** OA: octadecanedioic acid. Data were expressed as mean ± standard deviation followed by one-way ANOVA with Tukey's post-hoc test, n = 6 per group. **** $P < 0.0001$ versus control group.

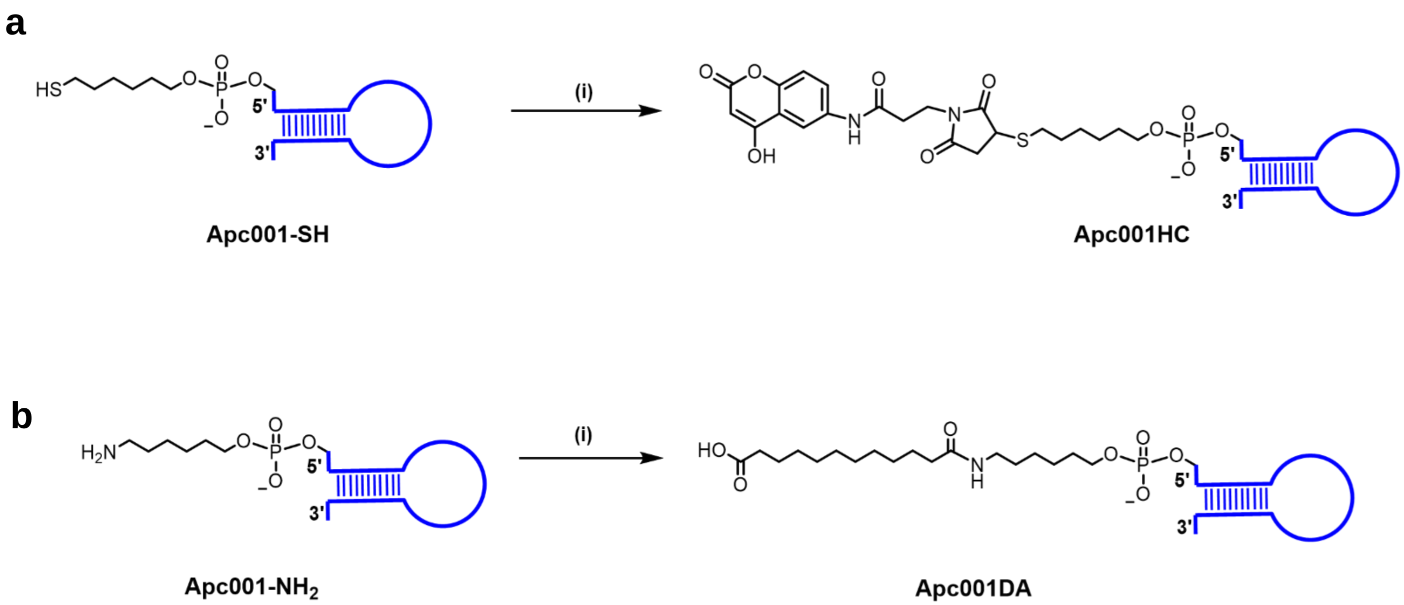

Figure S3. Synthesis of Apc001HC and Acp001DA. (a) Synthesis of Apc001HC**.** Note for reagents and conditions: (i) HC, H_2O/DMF, pH=6.75. **(b)** Synthesis of Apc001DA**.** Note for reagents and conditions: (i) DA, H_2O/DMF, pH=8.4.

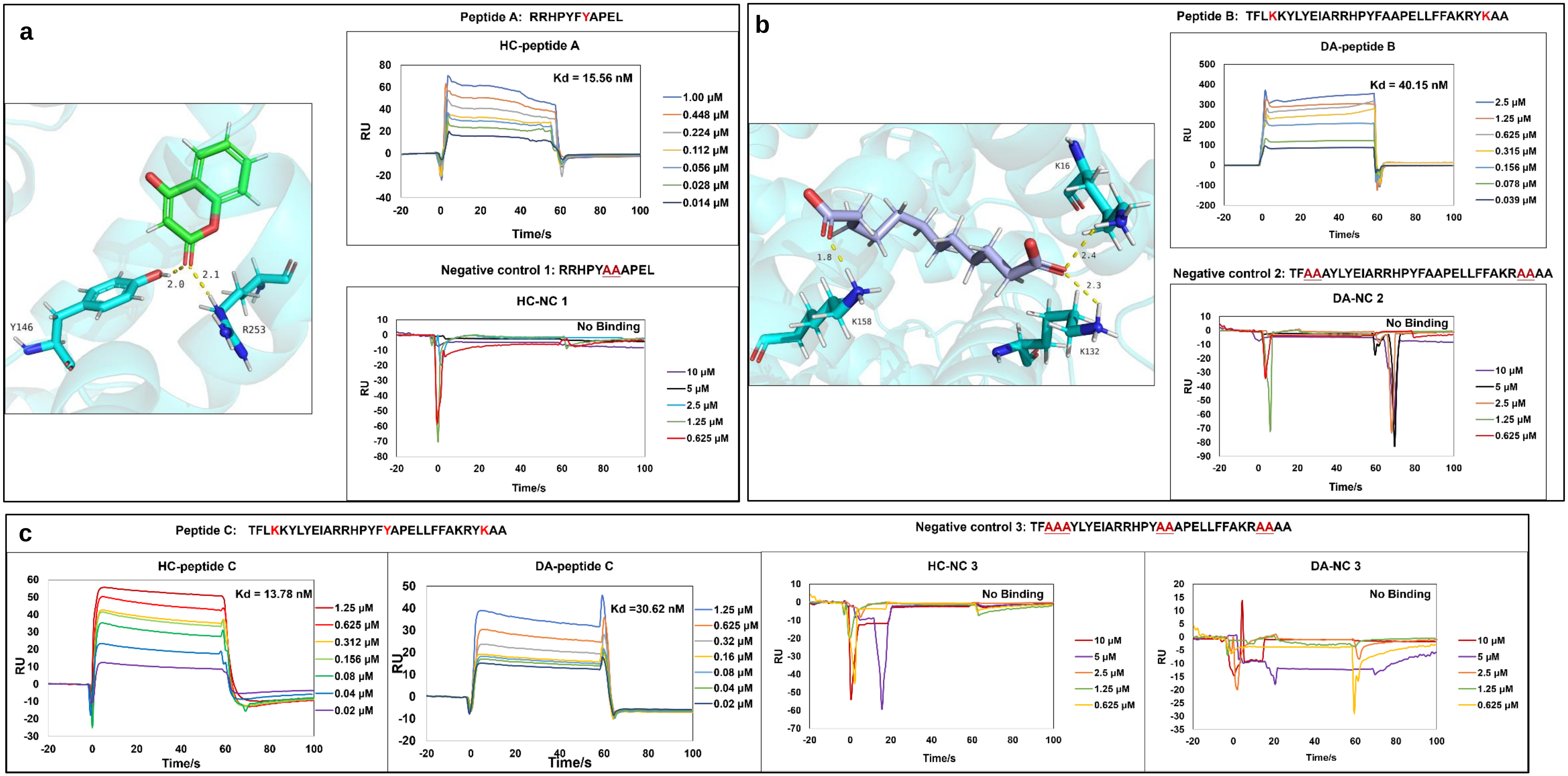

Figure S4. The design of the blocking peptide sequence which could block the binding of HC (peptide A), DA (peptide B) and HC-DA combination (peptide C) to HSA, respectively. (a) Left: Binding mode between the coumarin moiety of HC and HSA. Right: The sequences of peptide A / NC 1 and multi cycle kinetics analysis of binding affinity of HC to peptide A / NC 1. **(b)** Left: Binding mode between the fatty acid moiety of DA and HSA. Right: The sequences of peptide B / NC 2 and multi cycle kinetics analysis of binding affinity of DA to peptide B / NC 2. **(c)** The sequences of peptide C / NC 3 and multi cycle kinetics analysis of binding affinity of both HC and DA to peptide C / NC 3. **Note:** Based on the calculated binding sites of HSA to the coumarin moiety of HC, it was predicted that Y146 and R253 on HSA were involved. Then, we designed a peptide sequence (**peptide A**) including the above binding sites. Based on the calculated binding sites of HSA to the fatty acid moiety of DA, it was predicted that K16, K132 and K158 on HSA were involved. Then, we designed a peptide sequence (**peptide B**) including the above binding sites. Based on the aforementioned predictions, a peptide sequence including the above binding sites that could bind to HC-DA combination was designed **(peptide C).** We have designed the negative peptide sequences (NC1 for peptide A, NC2 for peptide B, NC3 for peptide C) by mutating the predicted binding amino acids sites to alanine on each blocking peptide. The amino acids in red represent the binding sites; NC: negative control. HC-DA combination: the HSA binding to both HC and DA.

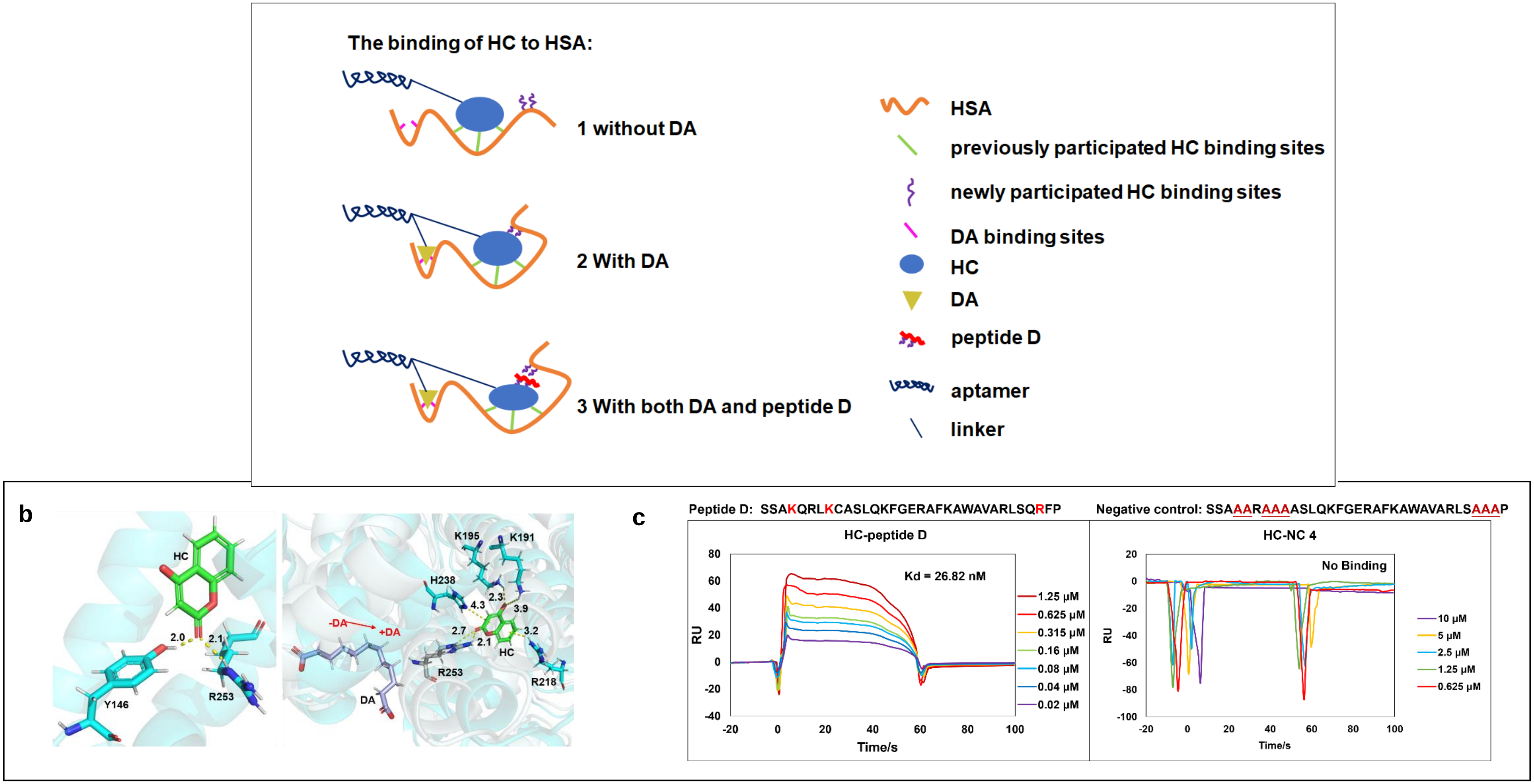

Figure S5. The design of the blocking peptide sequence which could block the binding of HC-DA interaction (by peptide D) to HSA. (a) Schematic diagram of blocking new sites of HC interacting with HSA at the presence of DA. **(b)** The binding mode between the coumarin moiety of HC and HSA, without the presence of the fatty acid moiety of DA (the left panel). The binding mode between the coumarin moiety of HC and HSA, with the presence of the fatty acid moiety of DA (the right panel). **(c)** The sequences of peptide D / NC 4 and multi cycle kinetics analysis of binding affinity of HC to peptide D / NC 4. **Note**: Based on the calculated binding sites of HSA to HC with and without the presence of DA, it was predicted that Y146 and R253 on HSA were involved without the presence of DA and K191, K195, R253, R238 and H238 on HSA were involved with the presence of DA. Interestingly, K191, K195, R238 and H238 were the newly participated binding sites with the presence of DA. Then, we designed a peptide sequence (peptide D) including the above newly participated binding sites and verified its binding affinity to HC. We have designed the negative peptide sequence (NC4) by mutating the predicted binding amino acids sites to alanine on peptide D. The amino acids in red represent the binding sites; 12C (colored in light blue): dodecanedioic acid; -12C: before molecular dynamic simulation; +12C: after molecular dynamic simulation; R253 (colored in gray): original residue, HC-DA interaction: the newly participated HSA binding sites to HC with the presence of DA.

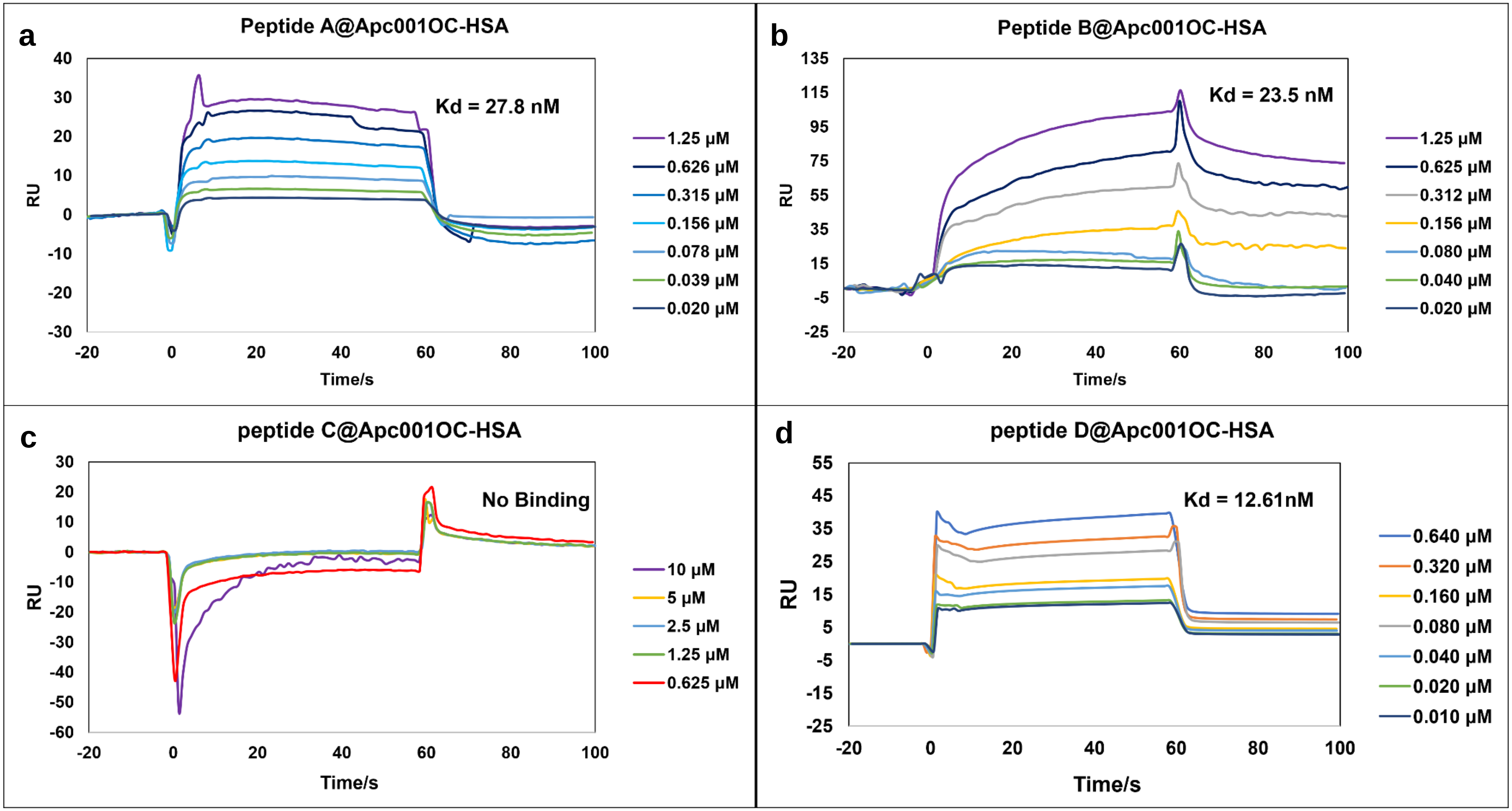

Figure S6. The binding affinity of Apc001OC conjugate to HSA was significantly reduced or abolished when it was pre-saturated with our designed blocking peptide sequences. (a) The binding affinity of the Apc001OC conjugate to HSA was significantly reduced when it was pre-saturated with peptide A. **(b)** The binding affinity of the Apc001OC conjugate to HSA was significantly reduced when it was pre-saturated with peptide B. **(c)** The binding affinity of the Apc001OC conjugate to HSA could be abolished when it was pre-saturated with peptide C. **(d)** The synergistically enhanced binding affinity of Apc001OC to HSA by DA conjugation was abolished when it was pre-saturated with peptide D. **Note:** HSA: human serum albumin. peptide A/B/C/D@Apc001OC: the blocking peptides-presaturated Apc001OC; peptide A: A peptide sequence within HSA included the predicted binding sites that could bind HC. peptide B: A peptide sequence within HSA included the predicted binding sites that could bind DA. peptide C: A peptide sequence within HSA included the predicted binding sites that could bind both HC and DA. peptide D: A peptide sequence within HSA included the predicted newly participated binding sites that could bind HC with the presence of DA.

a

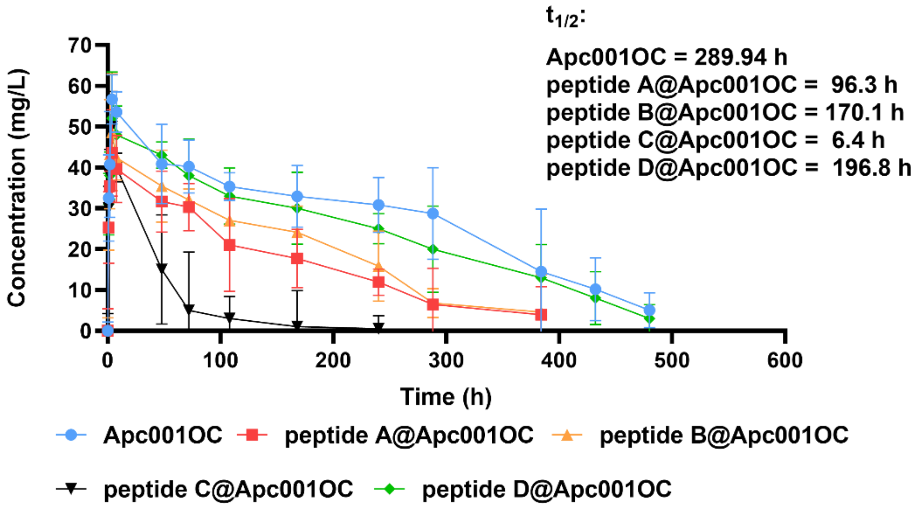

b

Parameter	Units	Mean value			
		peptide A@Apc001OC	peptide B@Apc001OC	peptide C@Apc001OC	peptide D@Apc001OC
T_{max}	(h)	4	4	4	4
C_{max}	(mg/L)	43	48	50	52
AUC	((mg*h)/L)	4230	6780	2256	9845
Elim. $T_{1/2}$	(h)	96.3	170.1	6.4	196.8

Figure S7. Pharmacokinetic analysis of Apc0001OC when it was pre-saturated with our designed blocking peptide sequences. (a) From the binding validation data, the peptide A, peptide B, peptide C and peptide D could block the binding of HC, DA, HC-DA combination and HC-DA interaction to HSA, respectively. The data demonstrated that the HC-DA combination effect on prolonging the half-life was reduced when it was pre-saturated with either peptide A (from $t_{1/2}$ =289.94 h to $t_{1/2}$ =96 h) or peptide B (from $t_{1/2}$ =289.94 h to $t_{1/2}$ =170 h). The HC-DA combination effect on prolonging the half-life was abolished when it was pre-saturated with peptide C (from $t_{1/2}$ =289.94 h d to $t_{1/2}$ =6 h). Importantly, the HC-DA interaction effect on prolonging the half-life was abolished when it was pre-saturated with peptide D (from $t_{1/2}$ =289.94 h to $t_{1/2}$ =196.8 h). **(b)** Pharmacokinetic parameters of peptide A@Apc001OC, peptide B@Apc001OC, peptide C@Apc001OC and peptide D@Apc001OC administered s.c, respectively. **Note:** peptide A/B/C/D@Apc001OC: the blocking peptides-presaturated Apc001OC; peptide A: A peptide sequence within HSA included the predicted binding sites that could bind HC. peptide B: A peptide sequence within HSA included the predicted binding sites that could bind DA. peptide C: A peptide sequence within HSA included the predicted binding sites that could bind both HC and DA. Peptide D: A peptide sequence within HSA included the predicted newly participated binding sites that could bind HC with the presence of DA.

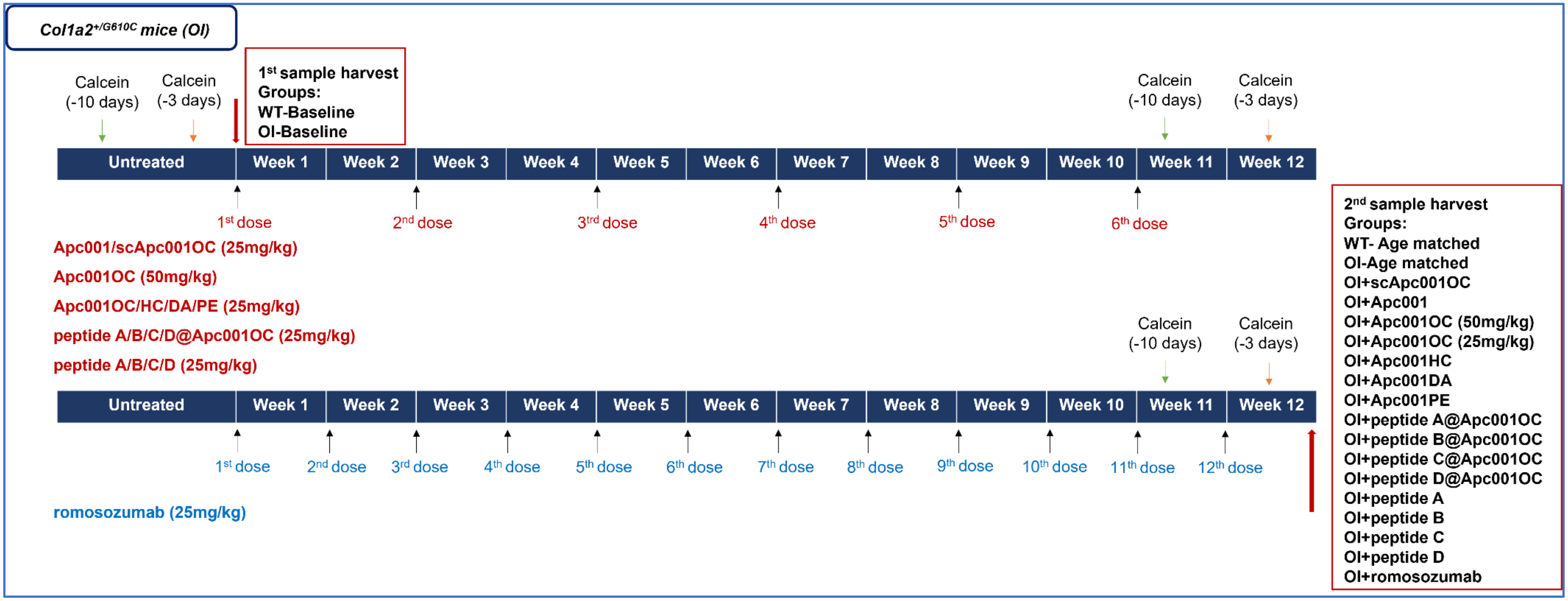

Figure S8. A schematic diagram showing the experimental design of the study. Briefly, six six-week-old *Col1a2*$^{+/G610C}$ mice (OI-Baseline) and six six-week-old wild-type littermates (WT-Baseline) were euthanized before treatment as baseline, respectively. Another six six-week-old *Col1a2*$^{+/G610C}$ mice (OI-Age matched) and six six-week-old wild-type littermates (WT-Age matched) were kept untreated for six weeks as the age matched groups, respectively. The remaining *Col1a2*$^{+/G610C}$ mice were subcutaneously administered with Apc001OC (25 mg/kg), Apc001OC (50 mg/kg), Apc001HC (25 mg/kg), Apc001DA (25 mg/kg), peptide A@Apc001OC (25 mg/kg peptide A + 25 mg/kg Apc001OC), peptide B@Apc001OC (25 mg/kg peptide B + 25 mg/kg Apc001OC), peptide C@Apc001OC (25 mg/kg peptide C + 25 mg/kg Apc001OC), peptide D@Apc001OC (25 mg/kg peptide D + 25 mg/kg Apc001OC), scrambledApc001OC (scApc001OC, 25 mg/kg), Apc001PE conjugate (25 mg/kg), non-conjugated Apc001 (25 mg/kg) or peptide A, B, C, D (25 mg/kg), respectively, once every two weeks for twelve weeks (n = 6 for each group), and romosozumab (25 mg/kg) once-weekly for twelve weeks (n = 6 for each group).

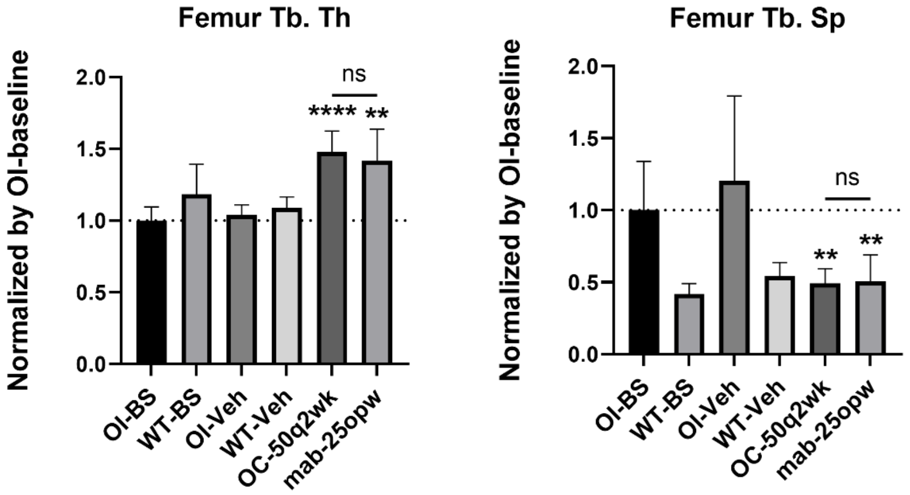

Figure S9. Bar charts of the structural parameters of Tb.Th and Tb.Sp from ex vivo micro-CT examination at the distal femur for the OI mice injected subcutaneously with 50 mg/kg of Apc001OC conjugate once every two weeks (q2wk) or 25mg/kg romosozumab for 12 weeks, respectively. Note: OC-50q2wk: 50 mg/kg Apc001OC conjugate q2wk; mab-25opw: 25 mg/kg romosozumab opw; Tb.Th: trabecular thickness; Tb.Sp: trabecular spacing; q2wk: once every two weeks; opw: once-weekly. Data were expressed as mean ± standard deviation followed by one-way ANOVA with Tukey's post-hoc test, n = 6 per group. ** $P < 0.01$; **** $P < 0.0001$.

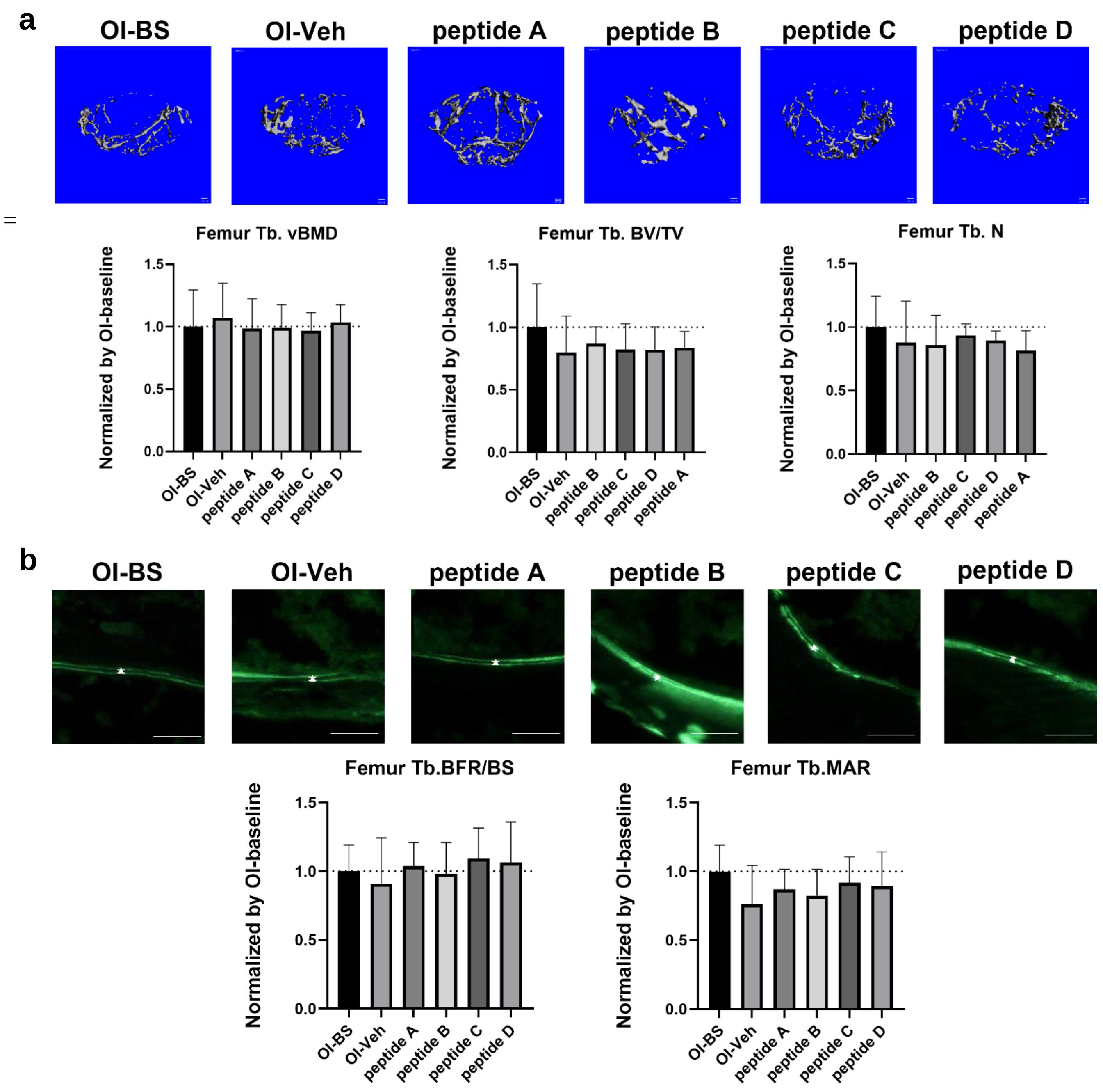

Figure S10. The blocking peptides had no effect on promoting bone formation, increasing bone mass and improving bone microarchitecture integrity at distal femur in OI mice. (a) Representative images showing three-dimensional trabecular architecture by micro-CT reconstruction at the distal femur. Scale bars, 0.1 mm (the upper panel). Bar charts of the structural parameters of Tb.vBMD, Tb.BV/TV and Tb.N from ex vivo micro-CT examination at the distal femur for the OI mice injected subcutaneously with 25 mg/kg of peptide A, peptide B, peptide C or peptide D once every two weeks (**q2wk**) for 12 weeks, respectively (the lower panel). **(b)** Representative fluorescent micrographs of the trabecular bone sections showing bone formation at the distal femur visualized by double calcein labels. Scale bars, 30 μm (the upper panel). Analysis of dynamic bone histomorphometric parameters of Tb.BFR/BS and Tb.MAR at the distal femur for the OI mice (the lower panel). **Note:** Tb.vBMD: trabecular volumetric mineral density; Tb.BV/TV: trabecular relative bone volume; Tb.N: trabecular number; Tb.BFR/BS: trabecular bone formation rate; Tb.MAR: trabecular mineral apposition rate; Peptide A: A peptide sequence within HSA included the predicted binding sites that could bind HC. Peptide B: A peptide sequence within HSA included the predicted binding sites that could bind DA. Peptide C: A peptide sequence within HSA included the predicted binding sites that could bind both HC and DA; Peptide D: A peptide sequence within HSA included the predicted newly participated binding sites that could bind HC with the presence of DA. Data were expressed as mean ± standard deviation followed by one-way ANOVA with Tukey's post-hoc test, n = 6 per group.

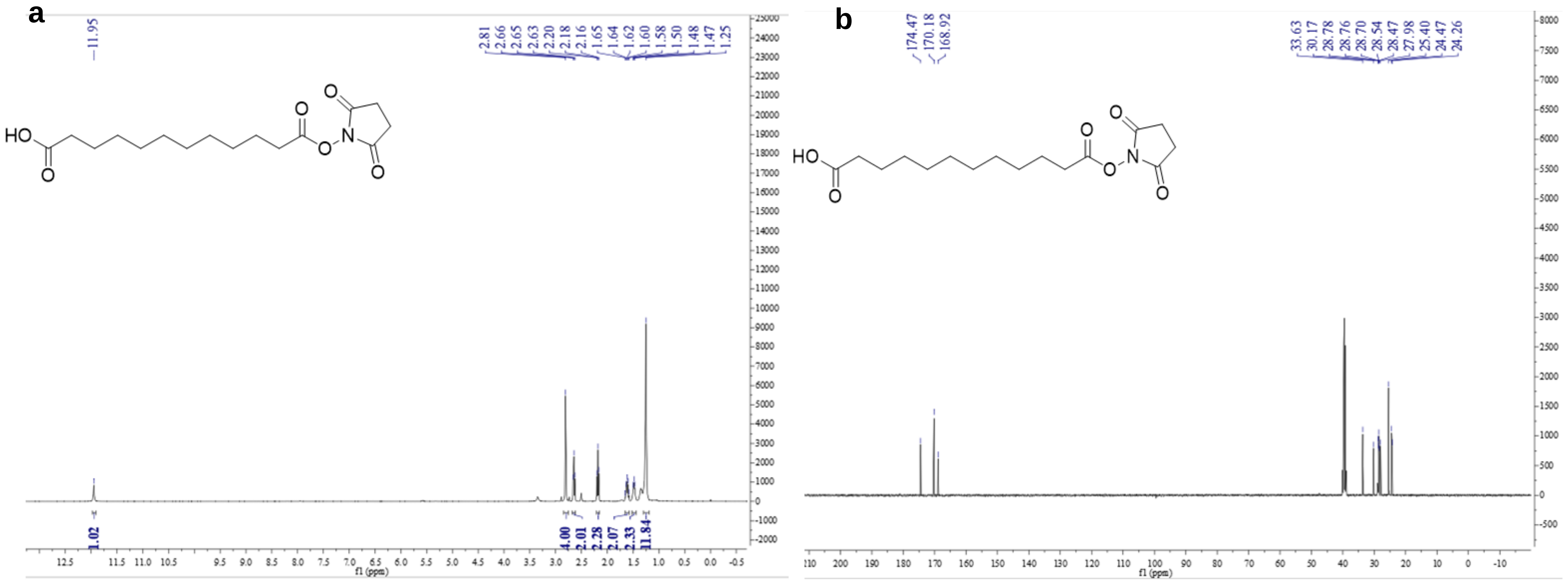

Figure S11. There were 25 protons in ^{1}H NMR spectra and 16 carbons in ^{13}C NMR spectra, respectively, which were matched the actual number of either protons or carbons of DA ($C_{16}H_{25}NO_6$). (a) ^{1}H NMR spectra. **(b)** ^{3}C NMR spectra. **Note**: **DA**: 12-((2,5-Dioxopyrrolidin-1-yl)oxy)-12-oxododecanoic acid. ^{1}H NMR (400 MHz, DMSO) δ 11.95 (s, 1H), 2.81 (s, 4H), 2.65 (t, *J* = 7.2 Hz, 2H), 2.18 (t, *J* = 7.4 Hz, 2H), 1.66 – 1.57 (m, 2H), 1.53 – 1.45 (m, 2H), 1.25 (s, 12H). ^{13}C NMR (101 MHz, DMSO) δ 174.5, 170.2, 168.9, 33.6, 30.2, 28.8, 28.8, 28.7, 28.5, 28.5, 28.0, 25.4, 24.5, 24.3.

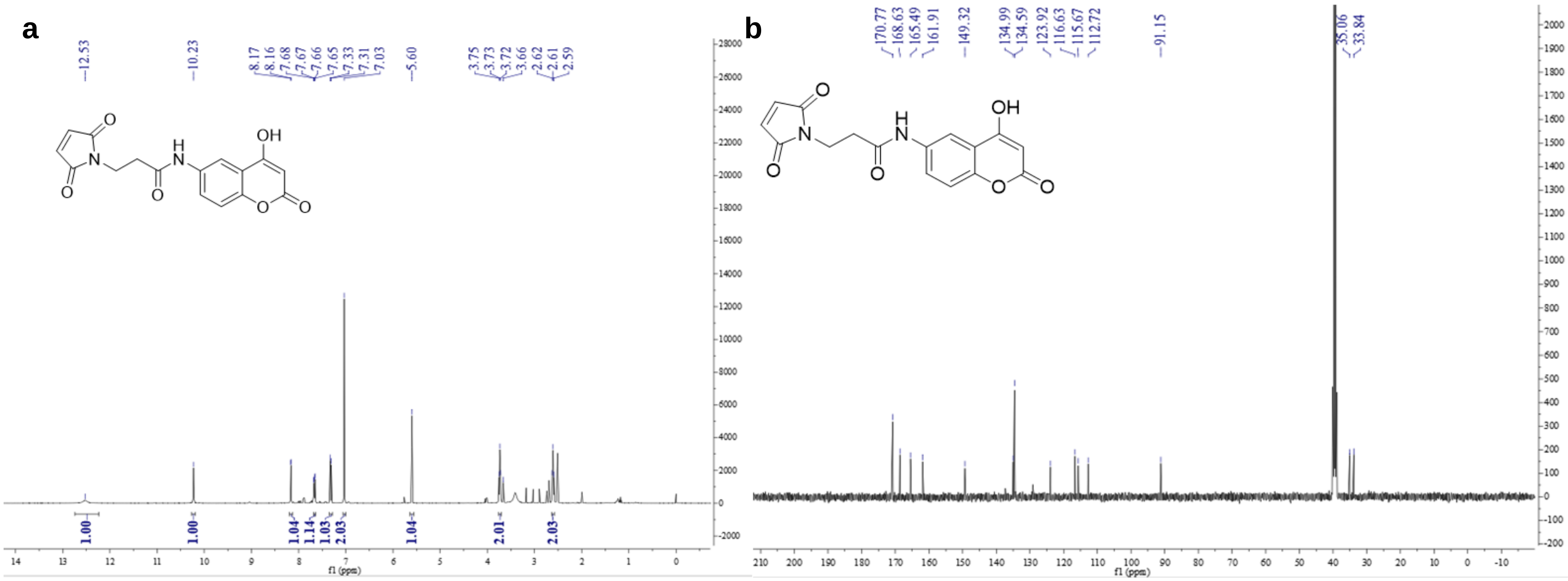

Figure S12. There were 12 protons in ^{1}H NMR spectra and 16 carbons in ^{13}C NMR spectra, respectively, which were matched the actual number of either protons or carbons of HC ($C_{16}H_{12}N_2O_6$). (a) ^{1}H NMR spectra. **(b)** ^{3}C NMR spectra. **Note**: **HC**: 3-(2,5-dioxo-2,5-dihydro-1H-pyrrol-1-yl)-N-(4-hydroxy-2-oxo-2H-chromen-6-yl)propenamide**.** ^{1}H NMR (400 MHz, DMSO) δ 12.53 (s, 1H), 10.23 (s, 1H), 8.16 (d, *J* = 2.5 Hz, 1H), 7.66 (dd, *J* = 8.9, 2.6 Hz, 1H), 7.32 (d, *J* = 8.9 Hz, 1H), 7.03 (s, 2H), 5.60 (s, 1H), 3.73 (t, *J* = 7.0 Hz, 2H), 2.61 (t, *J* = 7.0 Hz, 2H). ^{13}C NMR (101 MHz, DMSO) δ 170.8, 168.6, 165.5, 161.9, 149.3, 135.0, 134.6, 123.9, 116.6, 115.7, 112.7, 91.2, 35.1, 33.8.

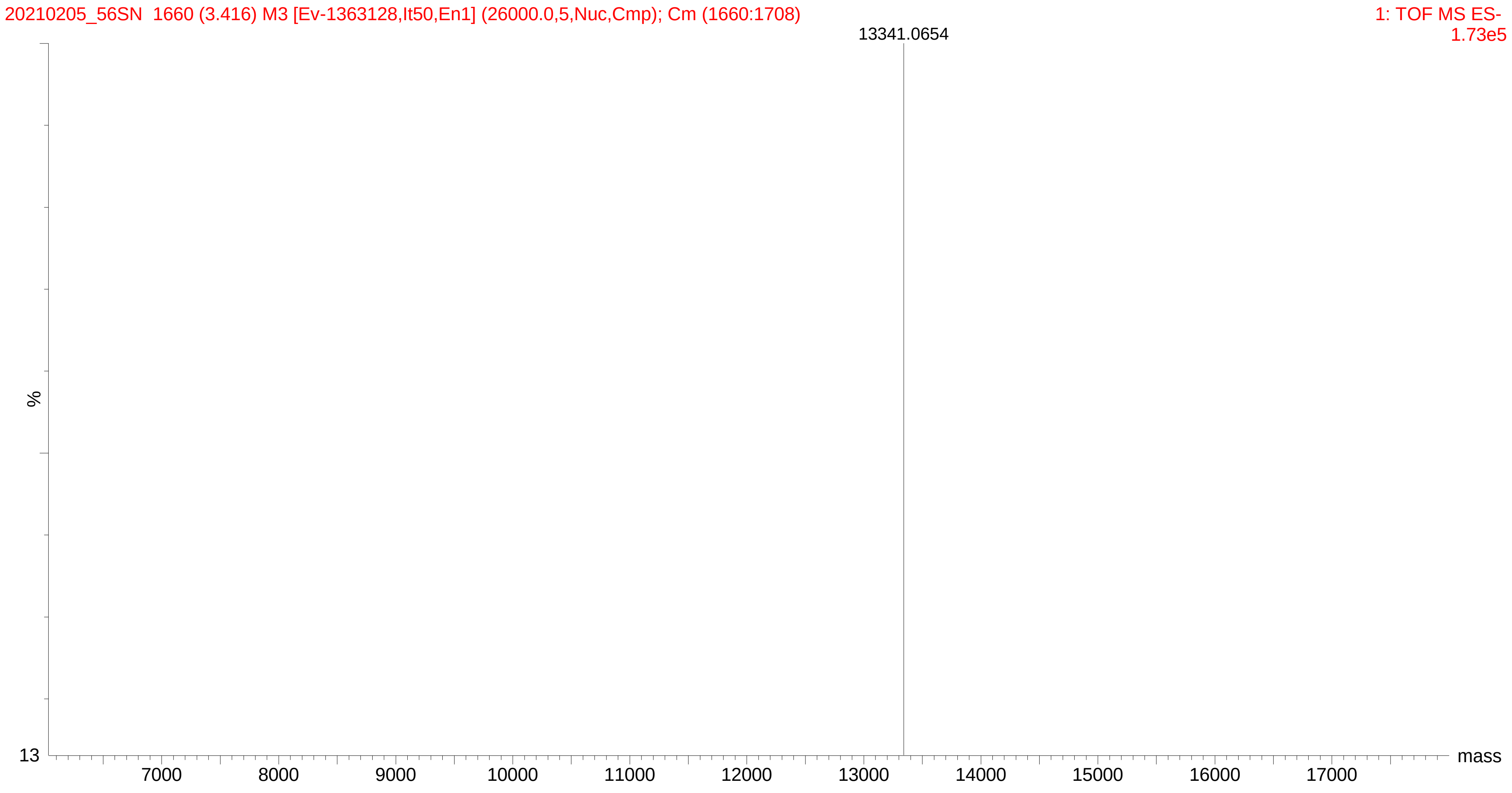

Figure S13. The mass of purified Apc001-SH-NH_2 was confirmed by ESI-Q-TOF-MS. Note: The actually measuring weight (13341.0654) matched the calculating weight (13348.7711).

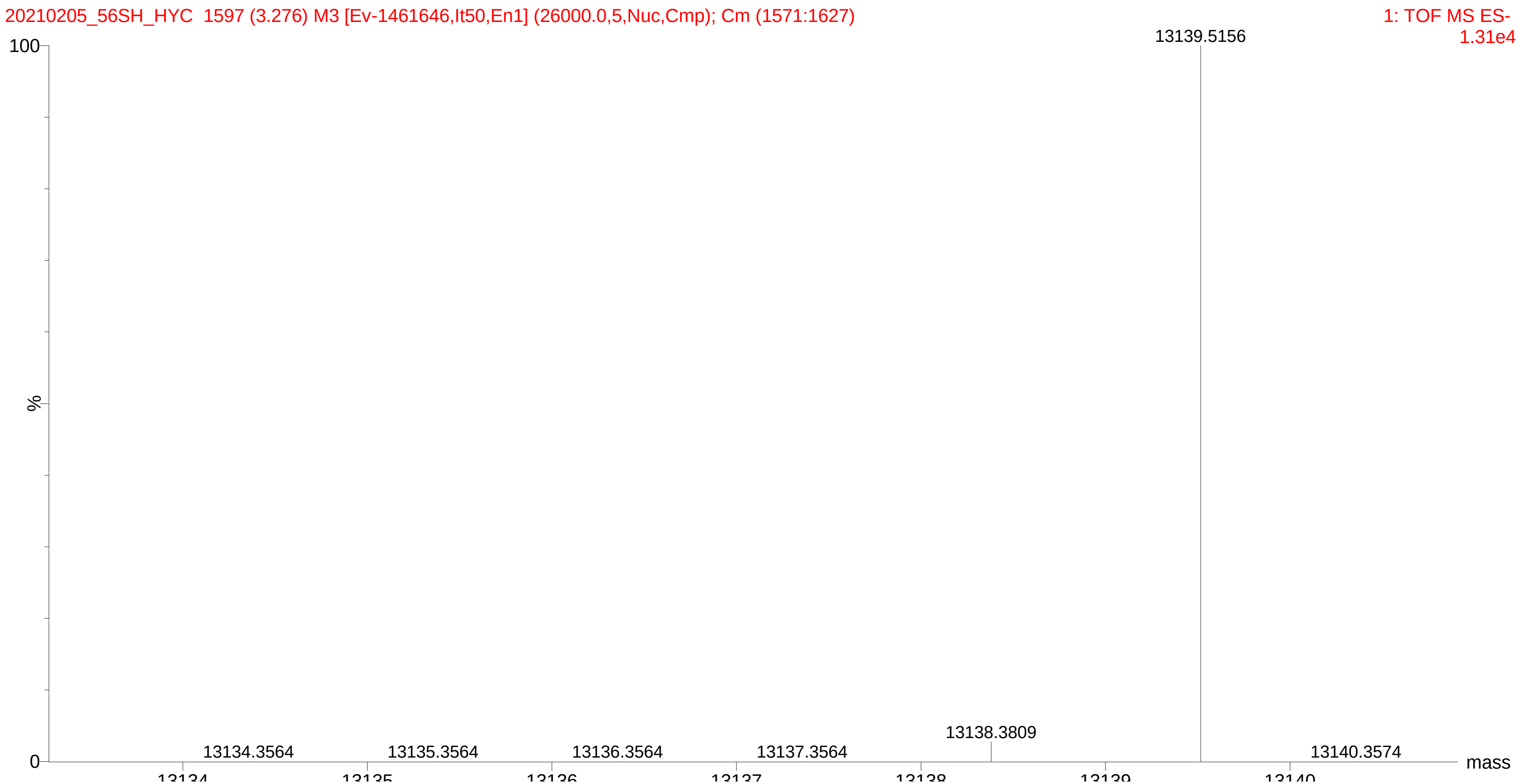

Figure S14. The mass of purified Apc001-SH was confirmed by ESI-Q-TOF-MS. Note: The actually measuring weight (13139.5156) matched the calculating weight (13139.5811).

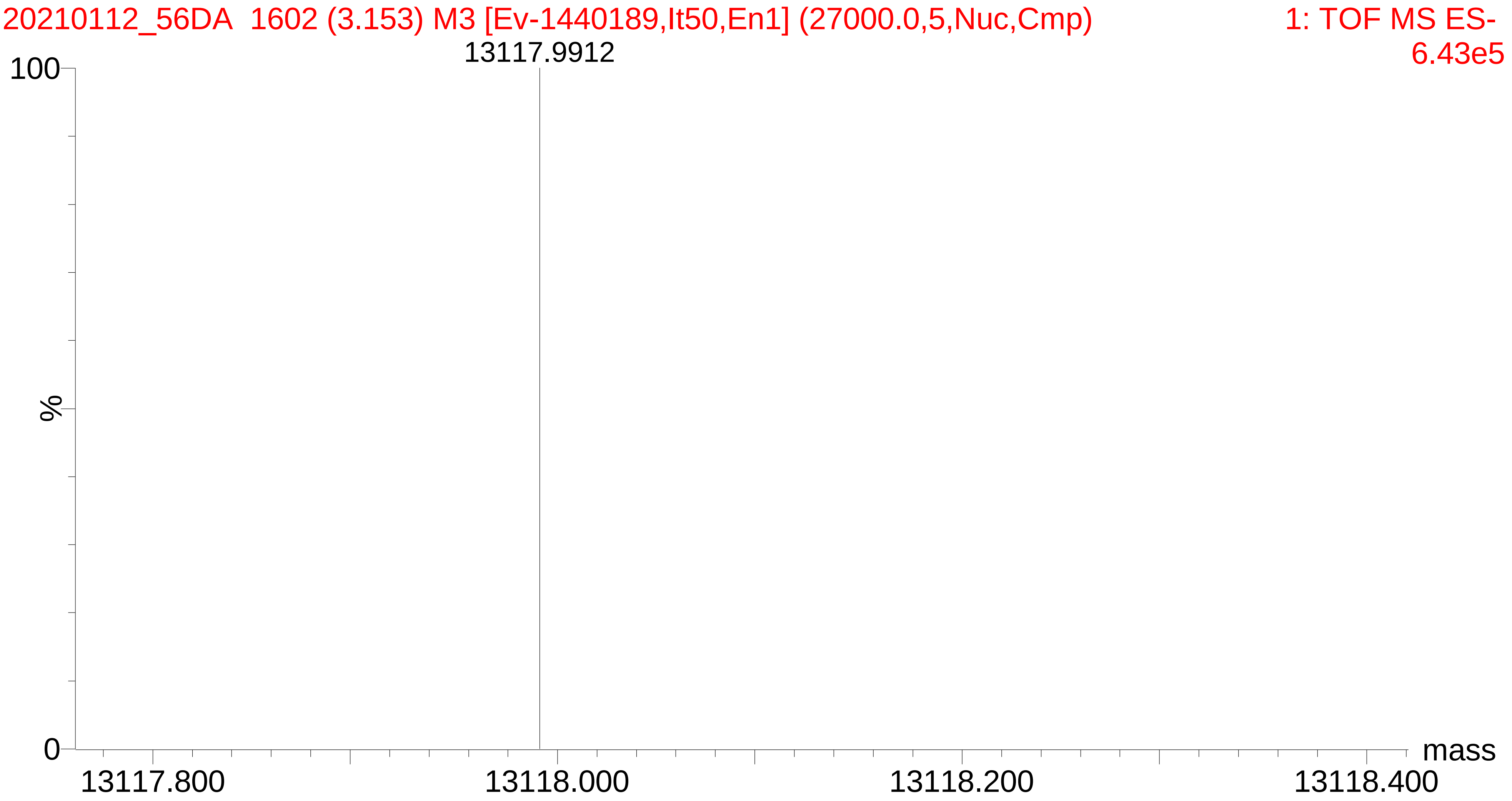

Figure S15. The mass of purified Apc001-NH_2 was confirmed by ESI-Q-TOF-MS. Note: The actually measuring weight (13117.9912) matched the calculating weight (13122.5415).

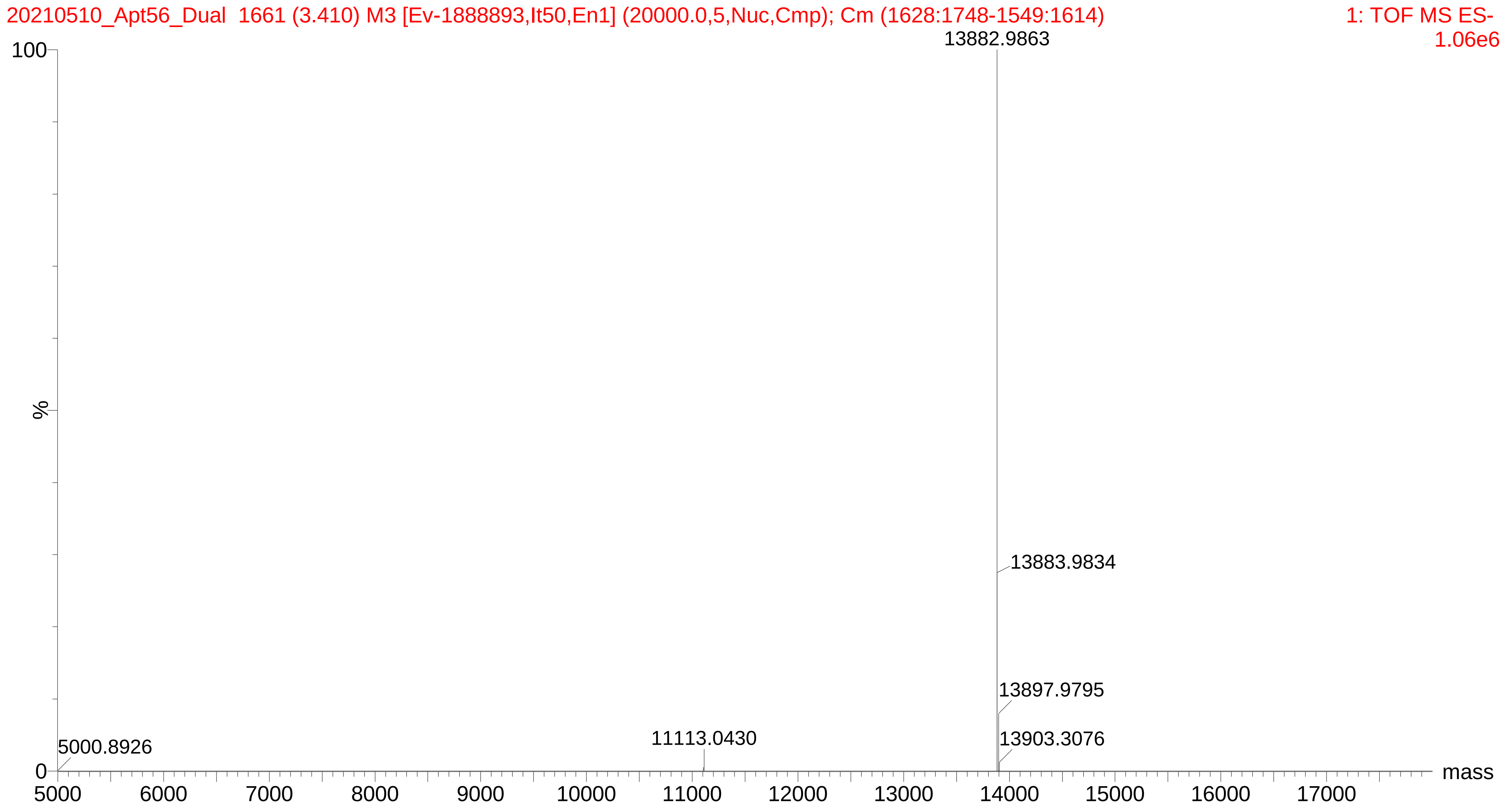

Figure S16. The mass of purified Apc001OC conjugate was confirmed by ESI-Q-TOF-MS. Note: The actually measuring weight (13882.9863) matched the calculating weight (13889.3463).

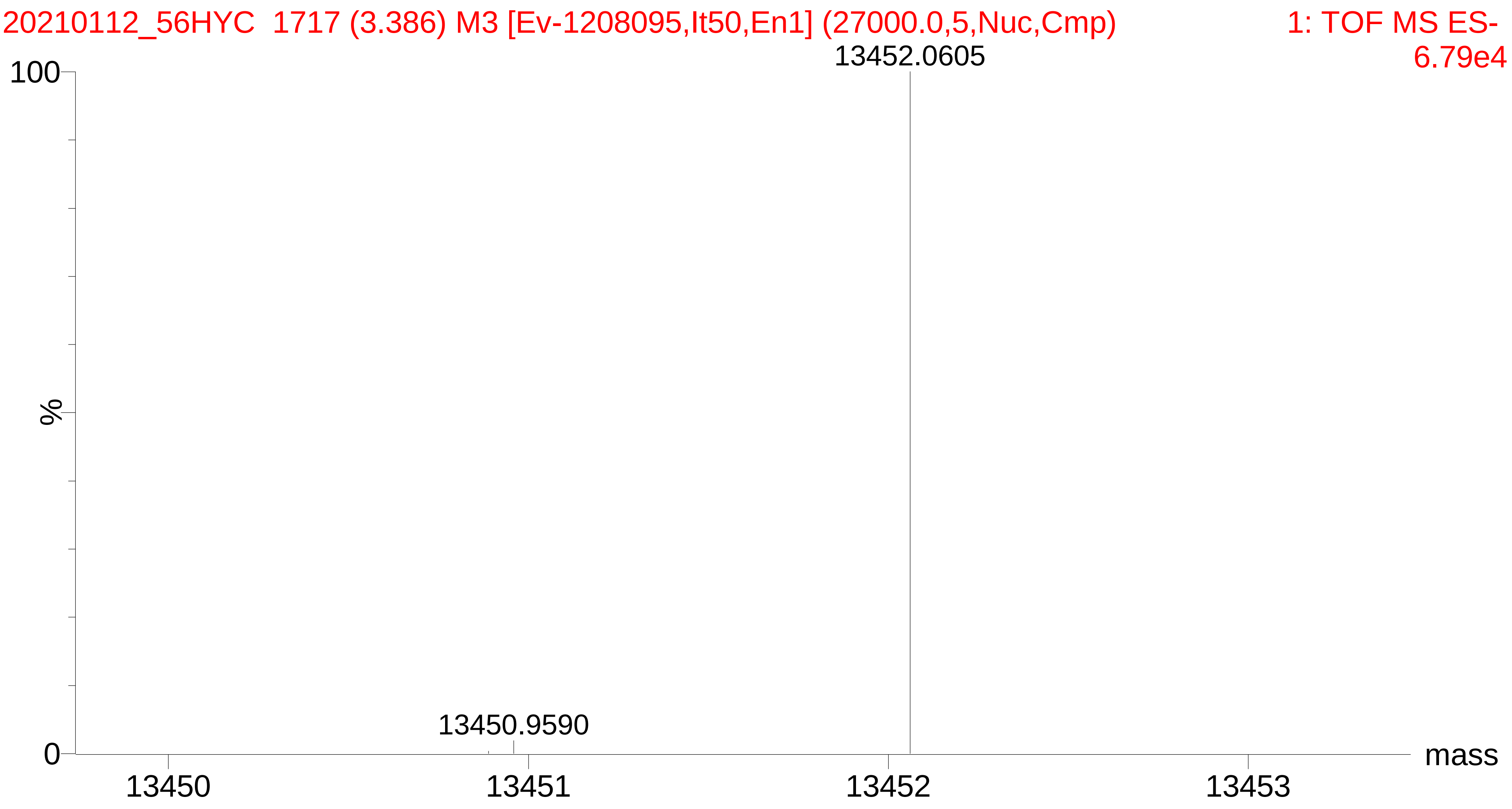

Figure S17. The mass of purified Apc001HC conjugate was confirmed by ESI-Q-TOF-MS. Note: The actually measuring weight (13452.0605) matched the calculating weight (13467.9685).

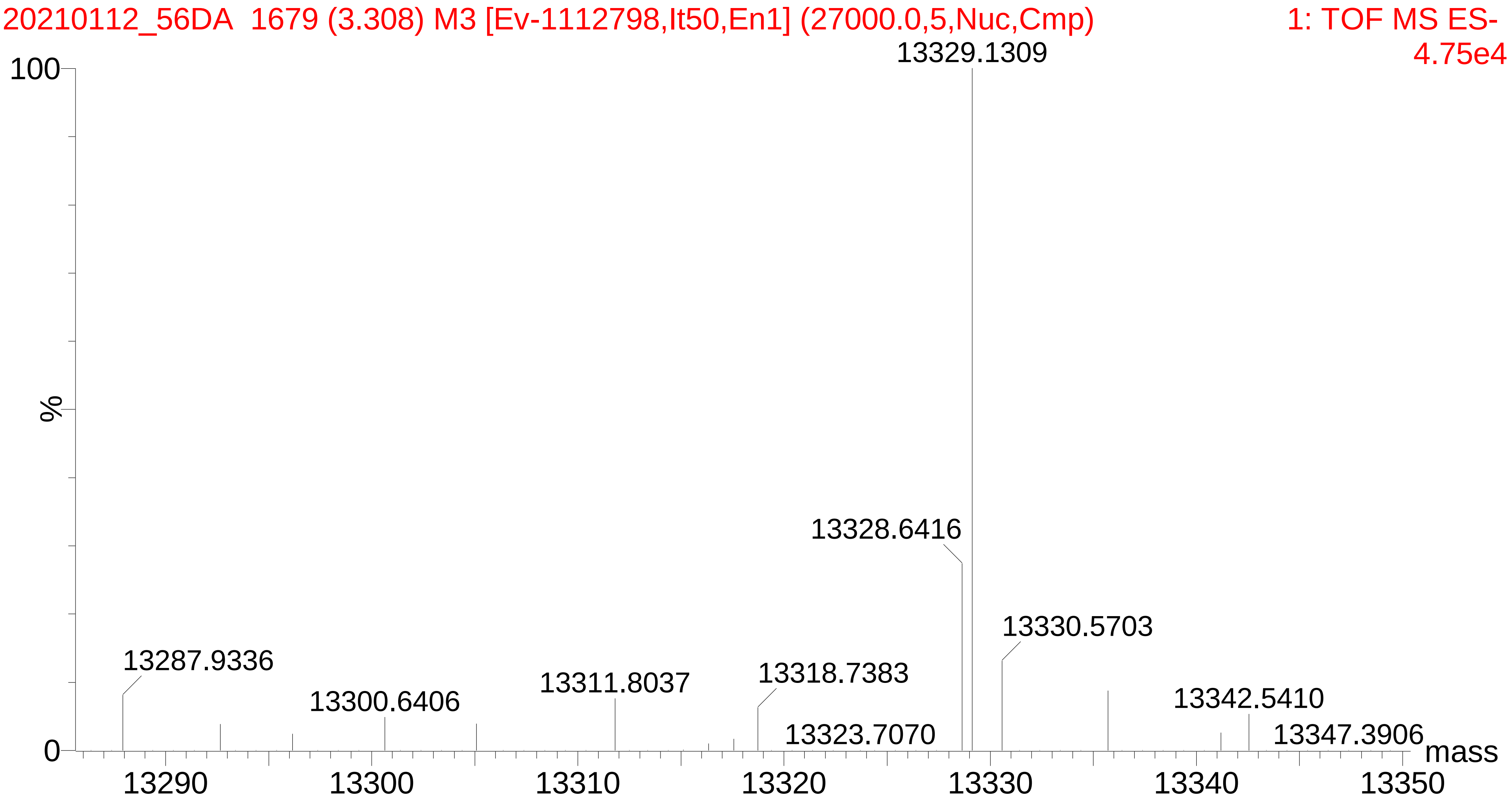

Figure S18. The mass of purified Apc001DA conjugate was confirmed by ESI-Q-TOF-MS. Note: The actually measuring weight (13329.1309) matched the calculating weight (13334.8250).

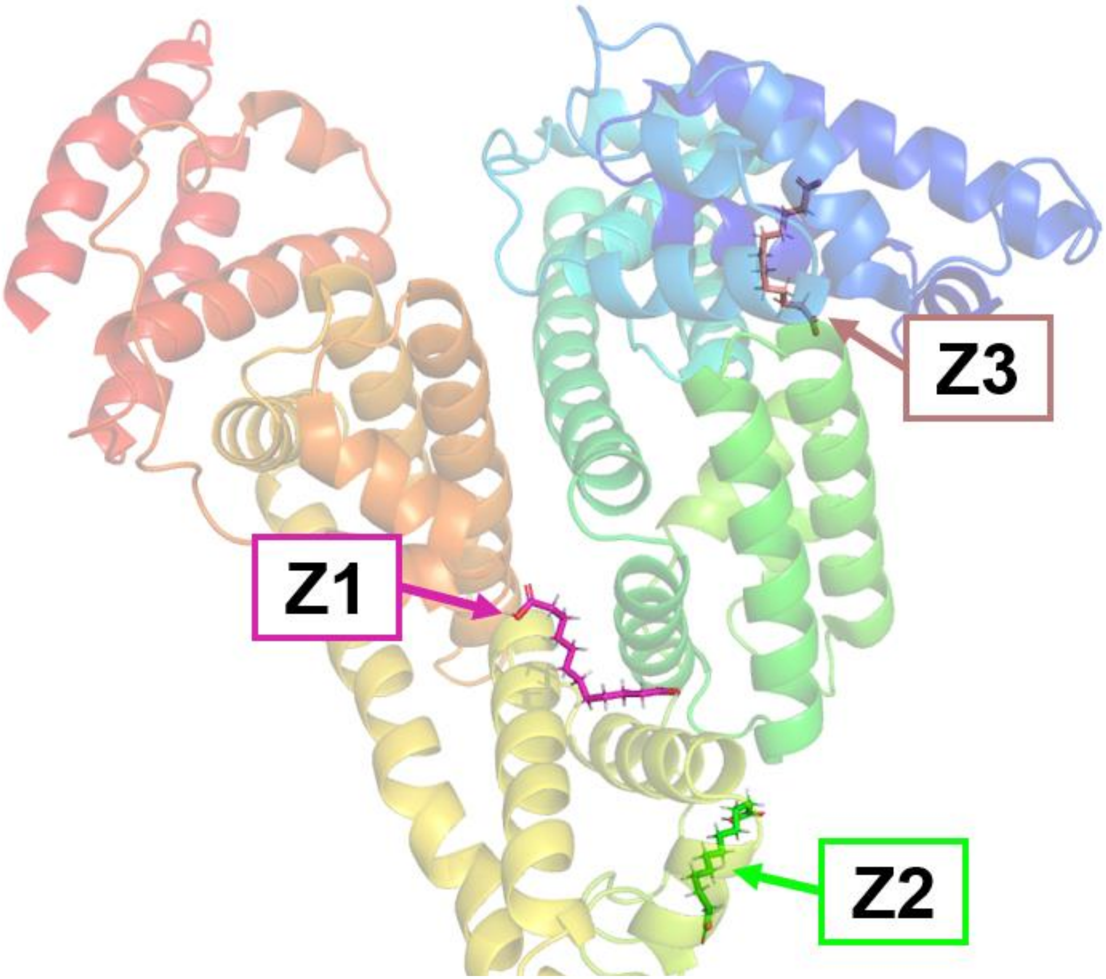

Figure S19. Three dodecanedioic acid (12C) binding pockets to human serum albumin (HSA). Note: The mentioned-above three 12C binding pockets to HSA were near the HC binding pocket to HSA, which was within the distance of 30 Å.

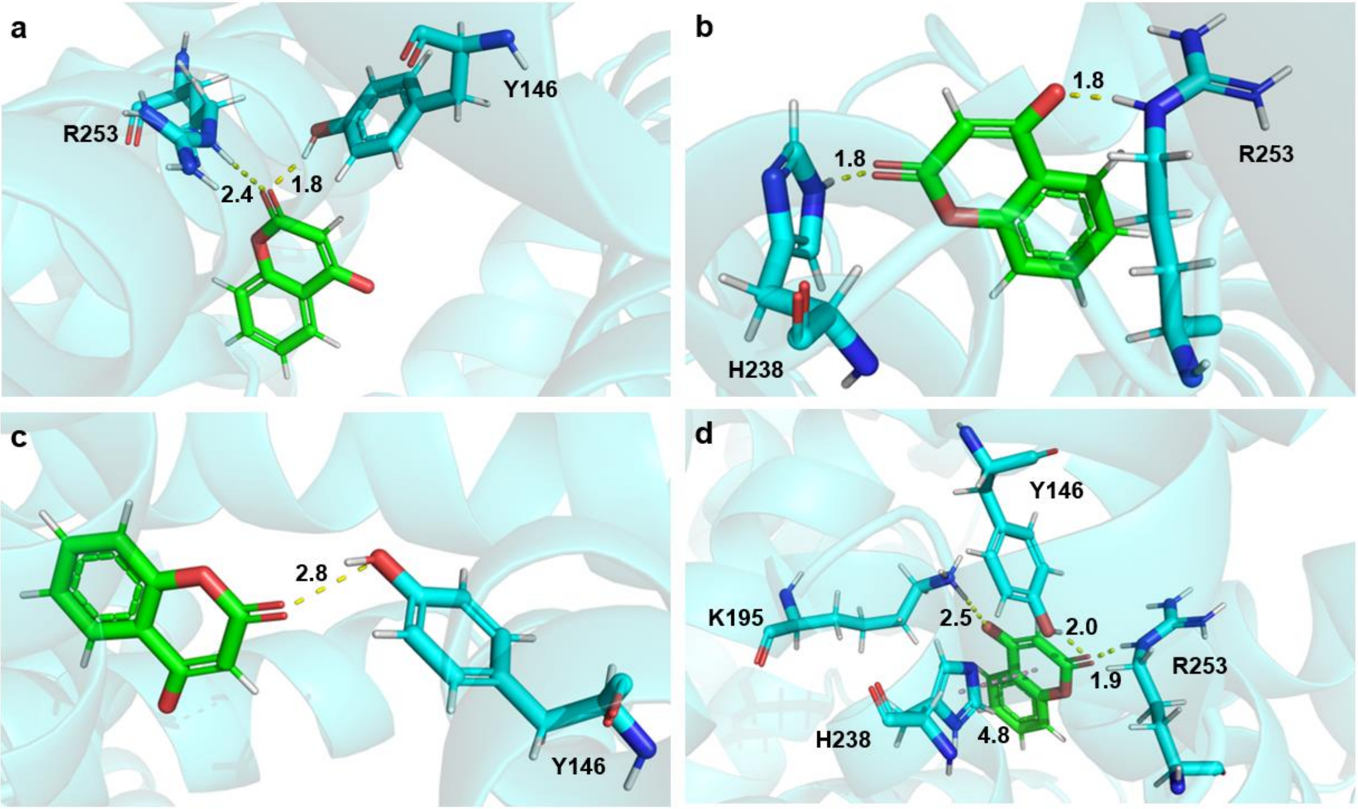

Figure S20. The binding modes between HC and human serum albumin (HSA) were predicted by interaction analysis, with/without DA in the pocket Z1, Z2, and Z3, respectively. (a) The predicted interactions between the coumarin moiety of HC and HSA, without the presence of the fatty acid moiety of DA near the binding pocket of HC to HSA, including two hydrogen bonds with Y146 (1.8 Å) and R253 (2.4 Å), respectively. **(b)** The predicted interactions between the coumarin moiety of HC (colored in green) and HSA, with the presence of the fatty acid moiety of DA in the pocket Z1, including two hydrogen bonds with H238 (1.8 Å) and R253 (1.8 Å), respectively. **(c)** The predicted interactions between the coumarin moiety of HC and HSA, with the presence of the fatty acid moiety of DA in the pocket Z2, including one hydrogen bonds with Y146 (2.8 Å). **(d)** The predicted interactions between the coumarin moiety of HC and HSA, with the presence of the fatty acid moiety of DA in the pocket Z3, including three hydrogen bonds with Y146 (2.0 Å), K195 (2.5 Å) and R253 (1.9 Å), respectively; and one Pi-Pi T-shaped interaction with H238 (4.8 Å). **Note:** K195 and H238 were the added predicted binding sites between the coumarin moiety of HC and HSA, with the presence of the fatty acid moiety of DA near the HC binding pocket to HSA**.**

a

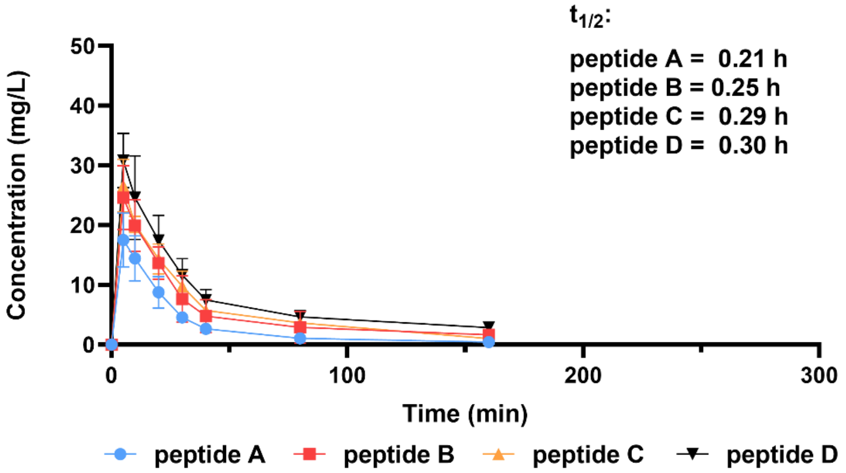

b

Parameter	Units	Mean value			
		peptide A	peptide B	peptide C	peptide D
C_{max}	(mg/L)	17.5	24.6	26.6	30.8
AUC	((mg*h)/L)	7.93	14.06	15.48	20.14
Elim. $T_{1/2}$	(h)	0.21	0.25	0.39	0.30

Figure S21. Pharmacokinetic analysis of peptide A/B/C/D in normal rats. (a) Pharmacokinetics of a single subcutaneous (s.c.) injection of peptide A/B/C/D (6.25 mg/kg) in normal rats. **(b)** Pharmacokinetic parameters of peptide A/B/C/D administered s.c, respectively. **Note:** Methodology referred to doi: 10.1038/ncomms16092.

Table S1 The binding affinity of Apc001, Apc001OC, Apc001HC and Apc001DA to HSA, respectively.

Aptamers	Kd value (Binding affinity to HSA)	Increased binding affinity to HSA compared to Apc001DA (%)
Apc001	No Binding	-
Apc001DA	46.6 nM	100%
Apc001HC	7.06 nM	660%
Apc001OC	0.75 nM	**6200% (＞760%=100%+660%)**

Table S2 The top eight coumarin derivatives without benzylacetone group with comparably high predicted binding affinity to human serum albumin (HSA).

Structure	Binding Energy (to HSA)	RSA_Ran k(to HSA)	Structure	Binding Energy (to HSA)	RSA_Rank (to HSA)
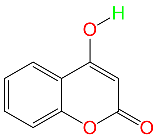 **4-Hydroxycoumarin**	**-6.91**	1	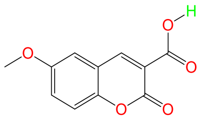 6-Methoxy-2-oxo-2H-chromene-3-carboxylic acid	-6.74	5
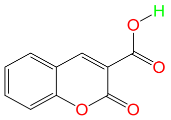 Coumarin-3-carboxylic acid	-6.89	2	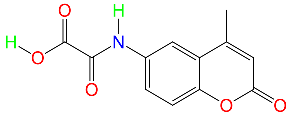 N-(4-Methyl-7-coumarinyl) oxalic acid amide	-6.57	6
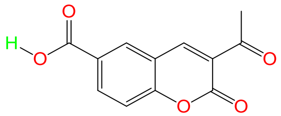 3-Acetyl-6-carboxycoumarin	-6.87	3	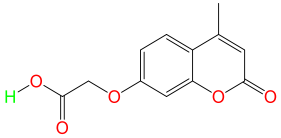 7-(Carboxymethoxy)-4-methylcoumarin	-6.55	7
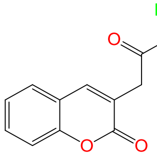 (2-Oxo-2H-chromen-3-yl) acetic acid	-6.82	4	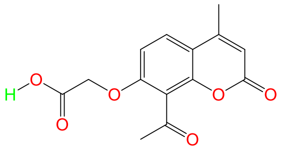 [(8-acetyl-4-methyl-2-oxo-2H-chromen-7-yl)oxy]acetic acid	-6.48	8

NOTE: Among all the tested warfarin derivatives, 4-Hydroxycoumarin had the highest binding affinity to HSA (PDB ID: 1AO6).

Table S3 The top nine fatty acids with comparably high predicted binding affinity to human serum albumin (HSA) and comparably low predicted binding affinity to fatty acid-binding proteins (FABP), respectively.

Entry	Structure	Binding Energy (to HSA)	RSA_Rank (to HSA)	Binding Energy (to FABP)	RSA_Rank (to FABP)
**18C**	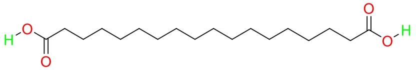	-4.88	1	-3.01	1
**12C**	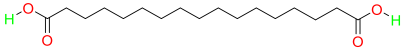	**-3.04**	**2**	**-0.14**	**5**
**16C**	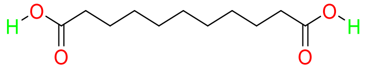	-1.26	3	-0.14	4
**17C**	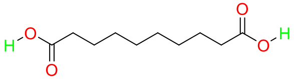	-1.26	4	1.38	6
**15C**	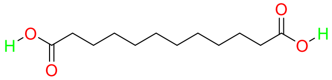	-1.19	5	7.16	9
**14C**	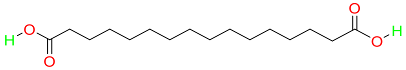	-0.70	6	6.70	8
**13C**	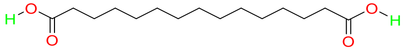	-0.64	7	6.60	7
**10C**	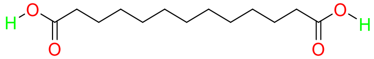	-0.62	8	-0.73	2
**11C**	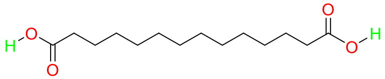	-0.06	9	-0.46	3

NOTE: 18C = octadecanedioic acid; 12C = dodecanedioic acid. Among all the tested truncated OA derivatives, dodecanedioic acid demonstrated the lowest predicted binding affinity (Energy: -0.23 kcal/mol, Rank: 1) to FABP (PDB ID: 1G5W) but comparably high predicted binding affinity to HSA (PDB ID: 1AO6).
